# Cellulose–Callose Hydrogels: Computational Exploration of Their Nanostructure and Mechanical Properties

**DOI:** 10.1021/acs.biomac.3c01396

**Published:** 2024-02-27

**Authors:** Pallavi Kumari, Pietro Ballone, Candelas Paniagua, Radwa H. Abou-Saleh, Yoselin Benitez-Alfonso

**Affiliations:** † The Astbury Centre and the Centre for Plant Science, School of Biology, University of Leeds, Leeds, LS2 9JT, United Kingdom; ‡ School of Physics and Astronomy, University of Leeds, Woodhouse Lane, Leeds, LS2 9JT, United Kingdom; § School of Physics, University College Dublin, Dublin 4 D04 C1P1, Ireland; ∥ Conway Institute for Biomolecular and Biomedical Research, University College Dublin, Dublin 4 D04 C1P1, Ireland; ⊥ Instituto de Hortofruticultura Subtropical y Mediterránea (IHSM-UMA-CSIC). Dpto. Botánica y Fisiología Vegetal, Universidad de Málaga, 29071, Málaga, Spain; # Department of Physics, Faculty of Science, Galala University, Galala Plateau, Attaka, Suez 43511, Egypt; ○ Department of Physics, Faculty of Science, Mansoura University, El Gomhouria St, El Mansoura 1, Dakahlia Governorate 35516, Egypt

## Abstract

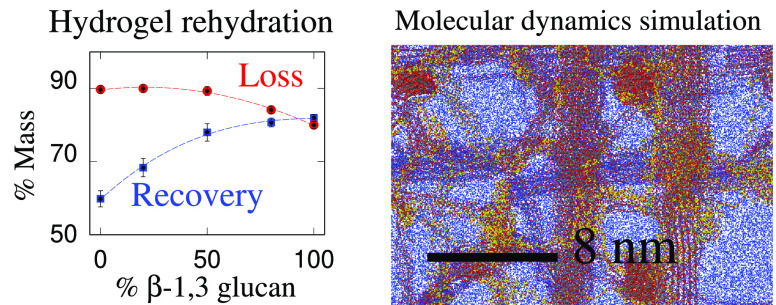

Polysaccharides play a crucial role in virtually all living systems. They also represent the biocompatible and fully sustainable component of a variety of nanoparticles, which are of increasing interest in biomedicine, food processing, cosmetics, and structural reinforcement of polymeric materials. The computational modeling of complex polysaccharide phases will assist in understanding the properties and behavior of all these systems. In this paper, structural, bonding, and mechanical properties of 10 wt % cellulose–callose hydrogels (β-glucans coexisting in plant cell walls) were investigated by atomistic simulations. Systems of this kind have recently been introduced in experiments revealing unexpected interactions between the polysaccharides. Starting from initial configurations inspired by X-ray diffraction data, atomistic models made of ∼1.6 × 10^6^ atoms provide a qualitatively consistent view of these hydrogels, displaying stability, homogeneity, connectivity, and elastic properties beyond those of a liquid suspension. The simulation shows that the relatively homogeneous distribution of saccharide nanofibers and chains in water is not due to the solubility of cellulose and callose, but to the formation of a number of cross-links among the various sample components. The broad distribution of strength and elasticity among the links implies a degree of anharmonicity and irreversible deformation already evident at low external load. Besides the qualitative agreement with experimental observations, the simulation results display also quantitative disagreements in the estimation of elastic coefficients, such as the Young’s modulus, that require further investigation. Complementary simulations of dense cellulose–callose mixtures (no hydrogels) highlight the role of callose in smoothing the contact surface of different nanofibers forming larger bundles. Cellulose–callose structures in these systems displayed an enhanced water uptake and delayed dye release when compared to cellulose alone, highlighting potential new applications as drug delivery scaffolds. The simulation trajectories provide a tuning and testing ground for the development of coarse-grained models that are required for the large scale investigation of mechanical properties of cellulose and callose mixtures in a watery environment.

## I Introduction

At first sight, the plant cell wall is primarily a protective envelop, confining the cytoplasm, offsetting the osmotic pressure from inside, imparting rigidity and providing support to cells and higher organisms.^1^ In reality, the plant cell wall plays a much broader and active role in the life of the cell, regulating the flow of nutrients and waste, implementing cell-to-cell communications, participating in cell replication and differentiation, and recognizing and opposing a number of pathogens. Cellulose is the plant cell wall main component, but other polysaccharide structures coexist in relatively large or small amounts. The dynamical character of the cell wall^2^ is emphasized by the fact that its composition and structure are species- and organ-specific and are finely regulated by a variety of signaling and gene expression processes, changing in time according to the different stages of cell life and in response to environmental stimuli.

Because of its abundance, relative simplicity and stability, cellulose played an early role in large-scale technologies such as the fabrication of natural textile fibers, paper, explosives, and more recently, the preparation of artificial textile fibers (Rayon). In the last decades, cellulose acquired a role as an engineering material^3^ and became a player in the burgeoning growth of nanotechnology,^4^ since cellulose nanocrystals of remarkably reproducible size and properties can easily be isolated from natural sources. Cellulose nanofibers, for instance, are of interest for drug delivery,^5^ energy harvesting and storage,^6^ and photovoltaic and photochemical devices.^7^ In all its applications, the appeal of cellulose greatly relies on its sustainability and low environmental impact.

The abundance of cellulose and its biological significance have obscured the role of a multitude of other chemical species, such as other polysaccharides, whose activity and regulation are strictly needed to carry out the variety of tasks fulfilled by the cell wall. Following an analytical approach, it is natural to extend the investigation of the cell wall by considering one by one further polysaccharides. The research reported here targets the (1,3)-β-d-glucan callose whose important functions in plant development and response to stress is widely recognized.^8^

Cellulose and callose share the same primary structure, both being β-glucans, differing in the location of the glycosidic bonds, which connect the (1–4) positions in cellulose and the (1–3) positions in callose. This seemingly slight change in the bond topology has important consequences. First of all, the tendency to molecular linearity, crystallization, and the formation of nanofibers, so characteristic of cellulose, are missing in callose.

The biological role of callose^8^ is emphasized by its unequal distribution among cells and along the cell wall, being particularly concentrated at plasmodesmata (intercellular channels in plants),^9,10^ at the pollen outer wall^11^ and at cell plates^12^ where the formation of a new wall is part of the cell replication process. In this way, the callose abundance and distribution alter the plasmodesmata permeability and thus transport and signaling among cells, the reproductive process of seed plants, as well as cell division. Callose is produced as helical chains,^13^ and the combination of cellulose and callose tends to give origin to 3D networks whose remarkable resilience probably underlies the accumulation of callose in response to biotic and abiotic stresses.^14^ It has been shown, for instance, that callose is the major component of papillae, i.e., rigid and thick patches on the surface of cells located at the site of fungal attack, whose likely role is to enhance the mechanical resistance of the cell wall.^15^ In its applications as a polymeric material, the tendency of (1,3)-β-glucans to helicity^16^ and to the formation of networks underlie its usage as a gelling additive.^17^

All the available information emphasizes the role of cellulose–callose combinations in the most dynamical aspects of the cell wall system, pointing to the interest of elucidating the interaction of these two related polysaccharides in complex environments and encompassing a range of size scales, from the atomistic to the mesoscopic domain of subcellular structures. To progress along these lines, a recent experimental study introduced a cellulose–callose hydrogel to isolate and thus characterize the interaction of cellulose and callose.^18^ Cellulose–callose mixtures (made using commercial chemical analogues) were dissolved in the 1-ethyl-3-methylimidazolium acetate ([emim][OAc]) ionic liquid (ILs), and hydrogels were prepared by replacing the ionic liquid with water. All the investigated systems consisted of 90 wt % water and 10 wt % polysaccharides, divided into a variable relative concentration of cellulose and callose from 0:100 wt % to 100:0 wt %. Besides morphology information, obtained from scanning electron microscopy (SEM) images and spectroscopy data, measurements focused primarily on mechanical properties of the hydrogels, including the Young’s modulus, the viscoelastic coefficients, and the plasticity index of all the samples. The main results concern the nonlinearity in the dependence of mechanical and viscoelastic properties on the relative cellulose–callose concentration. The conclusion is that specific, i.e., beyond mean-field, interactions are responsible for the observed nonlinearities, which, in turn, underlie the mechano-elastic properties relevant in the biology context.

These experimental data for relatively simple systems motivated the present computational investigation, whose aim has been to prepare hydrogel samples similar to the experimental ones, simulate them by molecular dynamics (MD) at the atomistic level, and provide a characterization of their properties that could allow molecularly explaining the experimental data. The simulation study has been preceded by a short experimental stage devoted to the X-ray diffraction (XRD) investigation of the hydrogel structure.^19^ Starting from an initial configuration reflecting the degree of crystallinity measured by X-ray diffraction, the atomistic simulation approach succeeds in producing a sample with the properties of a bona fide hydrogel. The simulation results also point to important nonlinearity in the stress–strain relation due to the presence of weak links among the polysaccharides. However, the results also show important quantitative discrepancies between the computed and measured values of the hydrogel Young’s modulus *Y*, that could be due to (i) differences between the hydrogel structure assumed by simulations and the one of the experimental system; (ii) significantly different times scales in the computational and experimental determination of *Y*.

A second, complementary simulation of cellulose bundles suggested that water uptake can improve when callose is added. The characterization of water and dye uptake/release, carried out by rehydrating a series of dried hydrogels whose composition and preparation parallel those of the samples investigated in the X-ray diffraction measurement, supports these predictions. These observations suggest directions to be followed in order to improve the present agreement between experiment and simulation, determining and validating along the way the interaction between cellulose and callose that drive the system behavior on the mesoscopic scale. It also highlights new properties of cellulose–callose mixtures that could inspire new applications in drug delivery systems and that improve our understanding of callose functions in plant cell walls.

## II Materials and Methods

### II.A Materials

Avicel PH-101 (cellulose) and the (1,3)-β-d-glucan Curdlan were purchased from Sigma Aldrich. The (1,3)-β-d-glucan Pachyman was purchased from Biosupplies Australia (www.biosupplies.com.au). Pachyman and Curdlan were used as callose commercial analogues. The ionic liquid 1-ethyl-3-methyl-imidazolium acetate [emim][OAc] (97% purity) was purchased from Sigma Aldrich. The cationic dye methylene blue (MB) was purchased from Sigma Aldrich and dissolved in Milli-Q water (Merck Millipore, Darmstadt, HE, Germany).

### II.B X-ray Diffraction Measurements on Hydrogels

Hydrogels were made as described in ref (18). In brief, Pachyman (or, when indicated, Curdlan) and Avicel were weighed and mixed at a 10% total polysaccharide in the ionic liquid [emim][OAc]. Samples were mixed and kept on a magnetic stirrer at 50 °C until dissolved. Water was added (and exchanged every few hours for 2 days) to remove the ionic liquid and form the hydrogels. Five samples were made, covering the full range of relative cellulose/callose wt % concentrations at the following discrete points: 100:0; 80:20; 50:50; 20:80; 0:100.

Wide angle X-ray diffraction (WAXD) was used to look at the changes in the peak pattern for all the gels, and to calculate the crystallinity index of the samples. Hydrogel samples were oven-dried overnight at 50 °C, XRD patterns for the background and the five samples were collected using the Huber 4-circle texture goniometer with CuKα radiation, generated at 40 KV and 30 mA, samples scanned over 2θ range between 10° and 40°, with a step of 0.10 and an interval of 180 s. The crystallinity index values were calculated using the ratio between crystalline and total areas, with the assumption that the increased amorphous contribution is the main reason for the peak broadening.^20^

### II.C Measuring Hydration Capacity of Composite Gels

Hydrogels were prepared as described in section II.B and in ref (18). To calculate relative differences in water uptake, the method published in ref (21) was followed. The initial weight of the gels was obtained (*M*_0_). Gels were dehydrated in an oven for 24 h or freeze-dried and weighed again (*M*_d_). The dehydrated gels were covered by water and allowed to rehydrate for 24 h. Excess water was removed by patting the gels in Whatman paper, and the rehydrated gels were weighed again (*M*_r_). The % mass loss was calculated as 100 × (*M*_0_ – *M*_d_)/*M*_0_. The % mass recovery was calculated as 100 × (*M*_r_/*M*_0_).

### II.D Dye Loading and Release

Methylene blue was dissolved in water at a concentration of 250 μg/ml. Several dilutions were prepared and absorbance was measured in a UV–visible spectrophotometer at 665 nm to obtain a calibration curve. The hydrogels prepared as described above were submerged in the methylene blue solution (250 μg/mL) for 24 h in a sealed bottle. After loading, the hydrogel was removed and the absorbance of the remaining solution was measured to calculate dye loading. The mass of the dye loaded was calculated using the calibration curve linear regression as *y* = 0.1913*x* – 0.0273; *R*^2^ = 0.9998 (where *y* = absorbance at 665 nm; *x* = dye mass loading). The calibration curve is given in Figure S1 of the Supporting Information (SI). To calculate dye release, the loaded hydrogel was submerged in 10 mL of fresh water. The absorbance of the aqueous solution was measured at 2 min intervals during 16 min. The release was calculated using absorbance and the calibration curve described above. The percentage of the dye release was calculated taking as a reference the dye loaded on the gel calculated before. The percentage of the dye release was represented and the slope of the linear part of the curve was referred to as rate release.

## III Computational Model

### III.A Force Field and Simulation Protocol

Computations have been carried out by molecular dynamics based on an atomistic force field of the Amber functional form.^22^ More precisely, the Gromos force field^23^ version 54a7^24^ has been used for modeling cellulose, callose, and to a limited extent, also [emim][OAc]. The solubility of organic molecules in the simple point charge (SPC) model of water^25^ has been used in ref (24) to tune the force field. For consistency, we used the same SPC water model in our simulations. The explicit parametrization has been obtained with the help of the *automated topology builder* ATB online utility.^26^ More precisely, we adopted a fragment-based approach, generating by the ATB Web site the force field parameter of a cellulose-type disaccharide, a callose-type trisaccharide and the single ions ([emim]^+^ and [OAc]^−^) of [emim][OAc]. The force field of the fragments have been joined to model polymeric chains and ions in water.

The investigated samples are contained in a orthorhombic (or cubic, as a special case) simulation box of sides *L*_*x*_ × *L*_*y*_ × *L*_*z*_, with periodic boundary conditions (pbc) applied. Long range electrostatic forces have been dealt with using the particle-mesh Ewald (PME) algorithm.^27^ Constant *T* conditions have been enforced using the Nosé-Hoover thermostat.^28,29^ Both canonical (NVT) and isobaric–isothermal (NPT) conditions have been used. Constant pressure, in particular, has been imposed using the Parrinello–Rahman^30^ barostat. At first, the fluctuations in the volume introduced to sample the NPT ensemble have been limited to isotropic changes that conserve the cubic shape of the simulation box. This choice limits fluctuations, speeding up the convergence of properties such as the average volume and the bulk modulus, but also prevents the determination of more general elastic constants. In the last stages of simulation, therefore, these constraints have been partly relaxed considering orthorhombic fluctuations of the simulation box, thus allowing the estimation of the *C*_11_, *C*_12_ elastic constants, and especially of the Young’s modulus *Y*, whose experimental value is available for the system under investigation.^18^ Molecular dynamics simulations have been carried out using the Gromacs package version 2019.^31^

The primary subject of the simulations are hydrogels made of a relatively dilute mixture of callose and cellulose (10 wt % total solute) in water. The dilute character of the systems, in turn, implies large samples and long simulation times. Hydrogel samples have been prepared by first inserting cellulose and callose polymers into the simulation box of a predetermined starting size, then filling the box with water using the solvate utility of Gromacs, reaching a system size of about 1.6 × 10^6^ atoms. After a first brief relaxation (∼50 ns) at NVT conditions, local equilibration lasting 200 ns at NPT condition has been performed to adapt the volume to the target pressure (*P* = 1 bar). Statistics has been accumulated over further 70 ns.

To summarize the simulation protocol, all simulations have been carried out at constant *T* = 300 K. Both NVT and NPT conditions have been used.

The analysis relies primarily on the visualization of trajectories, on the determination of the connectivity of the cellulose and callose network, and on the analysis of hydrogen bonding of cellulose, callose, water, and, in one case, of the [emim][OAc] ionic liquid. To characterize the different length scales relevant in hydrogel systems, suitable structure factors have been introduced. In particular, we computed separately the partial structure factor of water and of the carbohydrate. In both cases, a limited coarse graining has been carried out in computing the structure factor. In the case of water, for instance, only the water oxygens (OW) have been considered. Moreover, in the case of the polysaccharides, we computed the structure factor of the pyranose rings, each represented by its center of mass (com), computed by considering the five carbons and single oxygen in their 6-fold ring. No distinction between rings belonging to cellulose and callose is made at this stage. The computation and interpretation of the structure factors are discussed in more detail in the results section. Analysis of bonding is based primarily on a geometric definition of hydrogen bonds (HB, HBs). In the simulated systems, hydrogen bonds will concern O–H–O groups only (including those involving H_2_O), and triplets of this kind form a H-bond if the distance between the two oxygens is less than 3.2 Å and the deviation of O–H–O angle from linearity is less than 40°.

### III.B Structural Model of Cellulose and Callose Chains and Cellulose Nanocrystals

**Figure 1:**
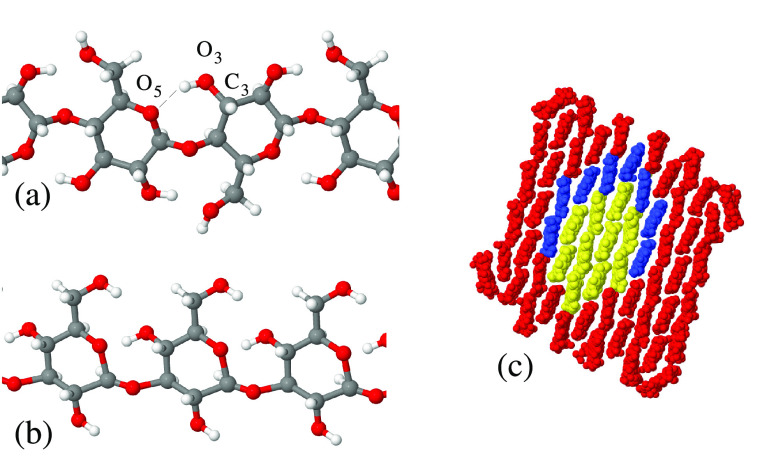
Atomistic structure of short segments of (a) cellulose and (b) callose chains simulated in the present study: gray dots, C; red dots, O; white dots, H. Both chains have been very briefly relaxed at *T* = 300 K during a few ps and do not represent equilibrium configurations. (c) Relation among the structure of the 10-, 18-, and 61-chain crystalline cellulose fibers illustrated through their cross section. The 10-chain core is painted yellow, and the 18- and 61-chain fibers are obtained by adding the cellulose chains painted in blue and in red, respectively. Irrespective of their color, all dots in (c) represent nonhydrogen atoms.

The organic molecular units of the simulated samples are represented by cellulose and callose polymeric chains (see Figure 1a,b), consisting of 16, 24, or 48 β-glucopyranose units. The details of the chain length and configuration of the two polysaccharide species depend on the properties targeted by each simulation and are listed in the Results and Discussion. In some cases, the simulated chains extend across the periodically repeated simulation box and lack terminations, thus representing infinitely long chains. These samples approach the chain structure of cellulose and callose in the plant cell wall, whose polymerization degree far exceeds the numbers achievable in a single simulation box. In other cases, as specified below, chains are of finite and relatively short length and are terminated at the two extremities by complementary −H and −OH groups. The terminations mitigate the rigid coupling of neighboring chain and nanofiber segments, bringing the mechanical properties of the simulated samples closer to those measured on the hydrogel systems.

The regular O3–H–O5 intrachain hydrogen bonding (see Figure 1a for the naming of the atoms) favors the linear configurations of cellulose chains, whose parallel arrangement gives origin to nanocrystalline fibrils that may be isolated from their amorphous matrix, for instance, by acid hydrolysis.^32^

The cellulose nanofibers that have been simulated have been carved out of an extended solid of Iβ crystal symmetry, and consist of 10, 18, or 61 cellulose chains. Their initial atomistic configuration has been prepared using the *cellulose builder* computer package documented in ref (33). The structural relation between the 61-, 18- and the 10-chain crystalline cellulose fibers is illustrated in Figure 1c.

Simulations of hydrogels and other cellulose–callose samples focus on the 10- and 18-chain cellulose nanofibers. The 18-chain cellulose-I nanofiber is generally considered the form of native cellulose.^34,35^ The 10-chain nanofiber is considered here as a model for the residual crystalline fraction of cellulose in hydrogels, following partial amorphization due to dissolution of nanofibers in ionic liquids, and successive replacement of ILs with water. Its size has been selected following an estimate of the crystallinity degree obtained from X-ray diffraction; see the results presented in section IV.A. The 61-chain nanofiber has been added to provide more data on the size dependence of cellulose nanofiber properties and, in particular, the size dependence of the interaction of callose single chains with cellulose crystal nanofibers. Admittedly, this size has no obvious biological relevance, but it could form as the result of postsynthetic processes, consisting of nanofiber aggregation and trimming.^34^ The approximate size of the nanofiber is 4.2 × 4.6 nm^2^, compatible with the wide range of sizes of square nanofibers listed in Table 2 of ref (32).

The relevance of these sizes is also due to the fact that they are delimited by relatively low energy surfaces. The 61-chain fiber, in particular, is delimited by four orthogonal (100) and (010) crystal surfaces. The arrangement of chains within the 18-chain nanocrystal has been selected, minimizing the potential energy of the relaxed (*T* = 0 K) geometry. The optimal (234432) planes arrangement agrees with that proposed in refs (36 and 37) and found in ref (38), and differs only slightly from the suggestion of ref (39). It is more rounded than the 61-chain nanofiber, being delimited by six narrow surfaces of the (110), (11̅0), and (200) type. Because of its small size, at *T* = 300 K, the structure of the 18-chain fiber (including its surfaces) is not as well defined as that of the 61-chain fiber. As expected, the crystalline ordering of the 10-chain nanofiber is less marked than in either the 18- or 61-chain nanofibers.

Besides cellulose nanofibers, cellulose single chains have been simulated as well, representing the noncrystalline cellulose fraction of hydrogels resulting from the dissolution process in [emim][OAc]. Callose chains do not have the same tendency to linearity and to the formation of nanocrystals. In the present study, they are modeled as single chains floating in water, freely interacting among themselves, with cellulose and ions, when present.

### III.C Validation of the Force Field of the Structural Models and of the MD Setup

In a preliminary validation stage, the structural and binding properties of cellulose and callose single chains in water were determined by simulating 48-ring long chains in 360 × 10^3^ water molecules over 50 ns. The large number of water molecules has been selected to prevent spurious chain self-interactions through periodic boundary conditions. On the other hand, the unfavorable statistics for a single chain, together with the sticky intrachain and chain-water H-bonding, trapping the sample into metastable configurations for relatively long times, prevent the quantitative determination of the average end-to-end separation in the two samples. Both cellulose and callose single chains in water form about 3.3 HBs per glucopyranose unit, with a 2:1 prevalence of bonds accepted from water with respect to those donated by the polysaccharide to water. In this solvated state, intrachain HBs are relatively rare, especially for cellulose, and at variance from the extended chains in crystalline nanofibers in most cases do not link consecutive glucopyranose units through the O3–H–O5 combination (mentioned in section III.A). For cellulose, the results are compatible with previous simulation data for single cellulose chains in vacuum and in water,^40−42^ as verified using simple scaling laws to compare results for different chain lengths.^43^ A similar study for callose has been carried out in the past,^44^ using a comparable model, simulated on a somewhat smaller scale (3 × 19 glucose units, 20 × 10^3^ water molecules, 700 ps). Also in this case, the previous results are compatible with those of the present simulation, although differences in the sample size and simulation time prevent a quantitative comparison. In a different context, (1,3)-β-glucan chains have been simulated together with proteins in docking studies,^45,46^ confirming that atomistic models like the one used in the present study provide at least a semiquantitative description of callose properties.

Again as a preliminary stage, the properties of the 10-, 18-, and 61-chain cellulose nanofibers have been investigated both in vacuum and in water suspension using the protocol described in section III. The results are presented in section S2 of the SI. In vacuum, the results highlight the important but not overwhelming role of intra- and interchain hydrogen bonding (HB), see also ref (47). In the 18-chain nanofiber, for instance, the average number of intra- and interchain HBs is about 1.5 and 0.4 per glucopyranose unit, respectively (see section S2 of the SI). As expected, the average value of intra- and interchain HB per glucose residue decreases slightly in going from the 18- to the 10-chain nanofibril and increases slightly in going to the 61-chain nanofibril, reflecting the lower relative role of surfaces and the enhanced stability of the crystal phase with increasing number of chains in the nanofiber. Cohesion of nanofibers, however, depends primarily on the dispersion energy (van der Waals) among the extended chains running parallel to each other (see computational data in section S2 of the SI). Adding water to the sample changes only slightly the intra- and interchain H-bonding of cellulose and results in the formation of water–cellulose HBs, primarily localized (as expected) on the (010) and (110) surfaces and virtually absent on the (100) surface. Once again, the number of these water–cellulose HBs is non-negligible in absolute terms, but low in relative terms, taking into account the size of the nanofibers. Also, in this case, quantitative data are given in section S2 of the SI. The results of this test stage of the simulation are consistent with those of ref (48). Data on surface properties of nanofibers computed with the same model are reported in ref (49).

The 10-, 18-, and 61-chain cellulose nanofibers are delimited by low-index crystallographic facets. Therefore, a preliminary assessment of nanofiber–callose interactions has been obtained by determining the adsorption energy and structure of callose on dry cellulose crystal faces, as reported in section S3 in the SI. Moreover, the same properties have been analyzed for single callose chains deposited on the dry crystal nanofibers, the results being reported in the same section S4 in the SI.

## IV Results and Discussion

### IV.A X-ray Diffraction Reveals Changes in the Hydrogel Structure

**Figure 2:**
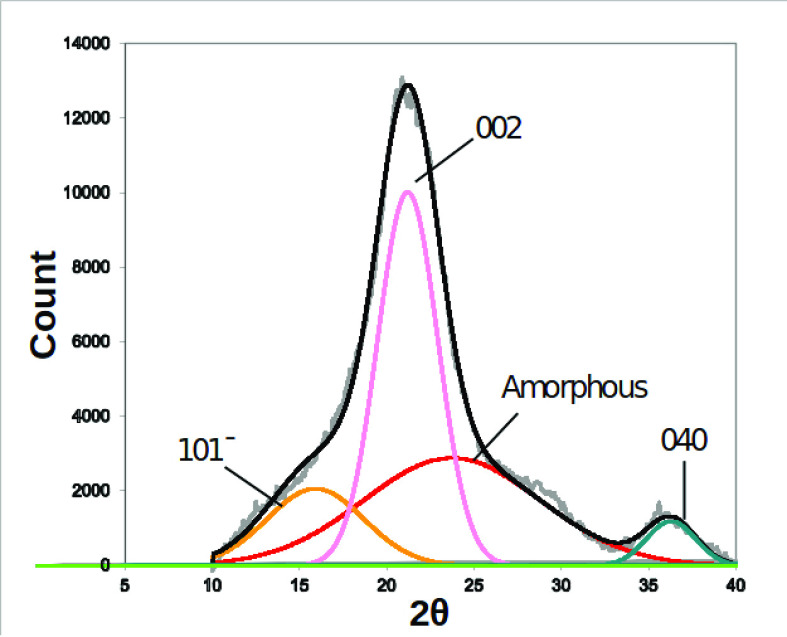
X-ray diffractogram of the dried cellulose hydrogel, showing an example of the peaks deconvoluted using the curve fitting process. The gray curve is the raw data, and the black curve is the fitting resulting from the peaks underneath. Peaks corresponding to crystallographic planes (101^–^), (040), and (002) and to amorphous cellulose are used to calculate the crystallinity index reported in Figure 3.

**Figure 3:**
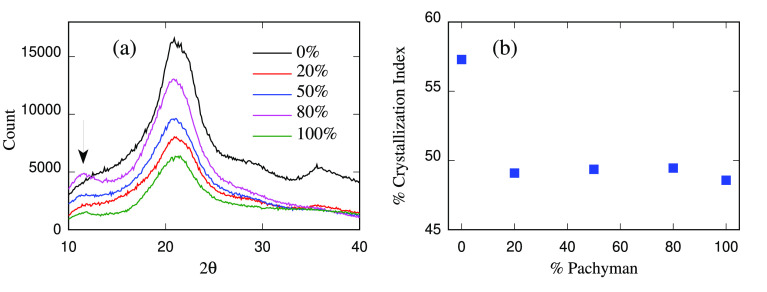
X-ray diffraction of cellulose–Pachyman hydrogels shows a decrease in crystallinity with increasing the % of Pachyman. (a) Raw diffractograms obtained from dried hydrogels containing different Pachyman concentrations (%). Arrows indicate a peak presumably associated with (1,3)-β-glucan structures. (b) Percentage of crystallinity was calculated by the area method from deconvoluted peaks using curve fitting process. A steep reduction is observed at the 20% Pachyman concentration, suggesting less-organized structures.

To gain structural information on cellulose/callose hydrogel structures, X-ray diffractograms were collected from a mixture of commercial cellulose/callose (Avicel:Pachyman) concentrations, as described in section II.B. The results are shown in Figure S7 of the SI. The diffractogram for the cellulose hydrogel shows a mix of peaks corresponding to different crystallographic planes (as indicated in Figure 2, see also ref (50)). A broad main peak at around 22° was detected for all the mixtures, including the 0% and 100% Pachyman concentrations (Figure 3). Other peaks contributing to the profile were identified using peak deconvolution as described before.^20,51^ As the Pachyman concentration increases, the crystalline peaks decrease. At the 80% sample, a new peak appears more visible at ∼12°, consistent with (1,3)-β-glucans, as described in previous research.^52^ The percentage of crystallinity was calculated with the peak deconvolution method,^53^ see also section II for further details. Figure 3B shows the highest value of 57% crytallinity for cellulose and a steep drop in the 20% Pachyman hydrogel sample. In comparison, the rest of the samples show small variations in this parameter. This last observation may cast doubt on the strict interpretation of the diffraction data, since crystal ordering persists in samples in which cellulose is disappearing or even disappeared (in the 0:100 wt % Pachyman). The focus of the present study, however, is on concentrations up to about 24 wt % Pachyman for which the interpretation of the diffractogram is adequate.

The X-ray diffraction results oriented the choice of the computational hydrogel samples toward a model in which cellulose is present as 10-chain crystalline nanofibers and single chains, these last accounting for the amorphous fraction. Conjecturing that the cellulose structure in the hydrogels results from the partial dissolution of the 18-chain nanofibers, the proportion of nanofibers and single cellulose chains has been set to one 10-chain nanofiber per eight single chains. Assuming that the nanofibers are ideally crystalline, this choice would correspond to a crystallinity degree of the cellulose sample (no callose) of 56%, similar to that calculated from X-ray data in the cellulose hydrogel.

### IV.B Preparation of Hydrogel Samples for Computational Studies

The main stage of the computational investigation consisted of the preparation and characterization of hydrogels made of cellulose and callose in water. As in experiments, the polysaccharide fraction accounts for about 10 wt % of the whole sample mass, but, in this constant amount, the relative concentration of cellulose and callose changes from sample to sample, as explained below. Since experiments do not provide a direct view of the microscopic structure of these systems apart from an indication of their crystallinity (60–40 wt % of crystal to amorphous ratio for the cellulose fraction), a few different models have been investigated, aimed at elucidating complementary aspects of all cellulose–callose mixtures in an aqueous environment.

**Figure 4:**
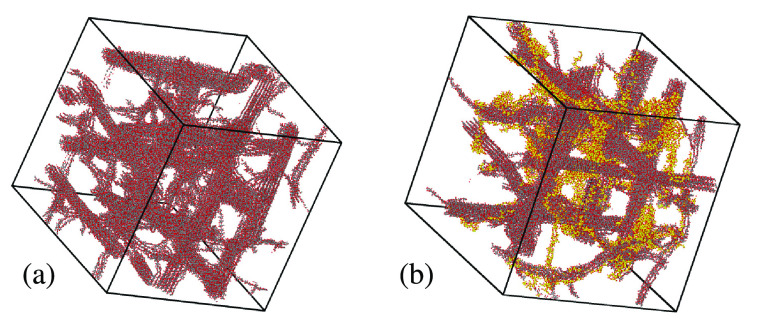
Perspective view of the hydrogel sample of (a) lowest and (b) highest callose concentrations considered in the present simulation study. Black dots: C atoms in cellulose; Yellow dots: C atoms in callose; Red dots: O atoms in either cellulose and callose. Water oxygens and all H atoms not shown. Both snapshots refer to the samples after a 250 ns relaxation time.

In the simulation, the preparation of hydrogels starts with a sample made of only cellulose (10 wt %) and water (90 wt %). In this first step, 12 cellulose nanofibers (10 chains each to mimic the crystallinity observed in the X-ray) have been inserted into a cubic box, divided into three groups of four equispaced and nearly parallel crystalline nanofibers directed along the *x*, *y*, and *z* directions, respectively. Additional 96 cellulose chains of approximately linear structure are added oriented in the same way, i.e., 32 chains along each orthogonal direction. Initially, these single chains are placed at random, with the constraint of leaving a minimum distance of 1 nm among themselves and from the nanofibers. To allow for the unconstrained reorientation of nanofibers and chains, all species are of finite length, with suitable terminations (as already stated in section III). All cellulose chains in the sample consist of 47 pyranose rings, whose length, in the ideal linear configuration of cellulose, is 24.4 nm. This fixes the initial length of the box to *L*_*x*_ = *L*_*y*_ = *L*_*z*_ = 25 nm. Together with a slight random tilt of the nanofibers and chains direction, this size is sufficient to prevent spurious interactions of chains with their periodic replicas. Then, each nanofiber and chain approximately aligned along *x* is shifted by a random fraction 0 ≤ *x*_rand_ ≤ 1 of *L*_*x*_ in the *x* direction. A similar random translation is applied to nanofibers and chains aligned along *y* and *z*. At this stage, the empty space in the simulation box is filled with water using solvate of Gromacs. In this way, the sample is homogeneous on the medium-large scale and is of approximate cubic symmetry, being equivalent along the three orthogonal directions (see Figure 4a). Moreover, as requested, the relative composition of water and cellulose is 90–10 wt %, while the 12 nanofibers and the 96 single chains account for the approximate 60–40 wt % crystal to amorphous ratio of the cellulose fraction of the system.

Three further samples were prepared, each time replacing one nanofiber and eight cellulose chains with the same amount (in wt %, corresponding also to the same number of replaced chains) of callose chains. In this way, the fraction of callose on the total polysaccharide content goes from 0 wt % to 8, 16, and 24 wt %, and the samples will be denoted as 100:0, 92:8, 84:16, and 76:24. Since the replacement is done one direction at a time, the 92:8 and the 84:16 samples deviate somewhat from the *xyz* symmetry, but the 100:0 and the 76:24 wt % samples retain the approximate cubic symmetry (see Figure 4b). The size and composition of the samples are listed in Table 1.

**Table 1:** List of the Major Simulated Samples^a^

sample	cellulose	callose	water
100:0	216 chains	0 chains	488821 molecules
92:8	198 chains	18 chains	487491 molecules
82:16	180 chains	36 chains	480305 molecules
76:24	162 chains	54 chains	473322 molecules
Bund-1	7 nanofibers	20 chains	0
Bund-2	7 nanofibers	20 chains	448 molecules
Bund-3	7 nanofibers	20 chains	26752 molecules

a In the first four samples, cellulose chains consist of 47 d-glucane units and callose chains are 48 d-glucane units long. Moreover, 56% of cellulose chains are grouped in crystalline nanofibers of a 10-chain cross section, while the remaining 44% is present as single chains. In the Bund-n case, described in section IV.E, cellulose chains are 16 d-glucane units long, callose chains are 24 d-glucane units long, and cellulose chains are joined in seven crystalline nanofibers, each consisting of 18 chains.

Assuming that our structural model of cellulose and callose chains dispersed in water is close to reality, several observations can be made by analyzing the simulation trajectories. First of all, the computational samples appear to be stable, and despite the poor solubility of cellulose and callose, no macroscopic phase separation of polysaccharides and water takes place. Moreover, cellulose nanofibers remain intact and nearly crystalline and neither shed their chains nor grow at the expense of the population of single cellulose chains. The initial approximate alignment of nanofibers along the axes changes only slowly with time, and, although somewhat altered, it is still recognizable after 300 ns. As expected, single chains, either cellulose or callose, drift more rapidly than nanofibers, and within about 20 ns they establish a number of links among themselves and with the cellulose nanofibers. It is important to remark that this way of representing crystalline and amorphous fractions is a plausible guess loosely based on experimental evidence. Even though the structure of natural microfibers has been extensively investigated, the mutual arrangement of their crystalline and amorphous fraction is not completely elucidated.^32,54,55^ The traditional view, possibly superseded by recent studies (see section 4 in ref (55)) is that, in their native state, cellulose nanofibers consist of crystalline and amorphous domains alternating along the main fiber axis.^54^ Therefore, another conceivable model could consist of 18-chain nanofibers in which crystal (56%) and amorphous segments (44%) alternate along the fiber axis. The first model has been preferred because the simulated fibers (24 nm long) are significantly shorter than the experimental ones (a few μm long) and dividing them into crystal and amorphous domains could destabilize their overall structure because of the relatively high weight of the interfacial free energy. Stating that the disordered sample made of cellulose, callose and water is indeed a gel and not simply a thick and viscous suspension is not trivial. The rigorous definition relies on the frequency-dependent viscoelastic properties of gels. These properties, however, are difficult to estimate by simulation, especially at the frequencies (∼kHz) of interest for comparing with experiments. Our discussion, therefore, will be more qualitative than quantitative, and will be based on elastic properties, as specified below.

### IV.C Structural Characterization of Hydrogel Samples

**Figure 5:**
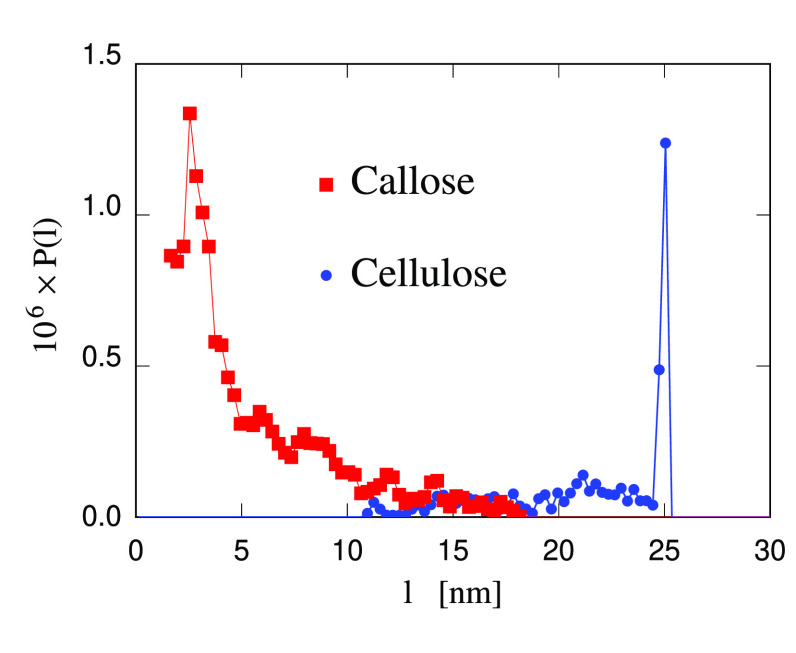
Probability distribution *P*(*l*) for the end-to-end separation *l* of cellulose and callose chains. The sharp peak at *l* = 25 nm is due to the fully extended cellulose chains in crystalline nanofibers, whose contribution is not included in the average ⟨*l*⟩ value quoted for cellulose. For each of the two species, 4π∫_0_^∞^*P*(*l*)*l*^2^ d*l* is equal to the number of chains belonging to that species.

The simplest structural characterization of the hydrogel samples concerns the end-to-end separation of cellulose and callose chains. This analysis finds that (as already apparent from snapshots) cellulose chains incorporated into the 10-chain nanofibers are fully extended, retaining a length close to the value for cellulose segments of the same number of pyranose rings in the Iβ crystal. Single chains in solution, either cellulose and callose, present a distribution of end-to-end separations. Probability distribution *P*(*l*) for the end-to-end separation *l* of cellulose and callose chains is defined in such a way that 4π*P*(*l*)*l*^2^ d*l* = *dN*(*l*), where d*N*(*l*) is the number of chains of the appropriate species α (the corresponding total number is *N*_α_) whose end-to-end separation is between *l* and *l* + *dl*. Due to several chain–chain and chain–nanofiber links that stabilize the hydrogel, 100 ns after the sample preparation the distribution of end-to-end distances in cellulose and callose single chains is virtually frozen. At this stage, the distribution of end-to-end separations for callose covers a narrower range than for single cellulose chains, the average separation being ⟨*l*⟩ = 20.2 ± 0.2 nm and ⟨*l*⟩ = 10.3 ± 0.2 nm for cellulose and callose single chains, respectively, measured on the 76:24 sample (see Figure 5). In the cellulose case, the quoted average does not account for the contribution from the fully extended chains in microfibers.

The difference in ⟨*l*⟩ certainly reflects the different persistence length of cellulose and callose in water, but because of the observed irreversibility of the links formation, there is no assurance that the distribution of end-to-end separation measured by simulation corresponds to equilibrium. This also implies that the statistical error bar quoted on ⟨*l*⟩ might neglect a systematic contribution due to the dependence of the result of the sample preparation and history. It is also important to remark that, despite the drastically different time scale of simulation and experiments, the same metastability of the cellulose and callose network in water might also affect the properties of the experimental samples.^56^

At constant water–polysaccharide relative concentration, the structure of the polysaccharide network, determined by the number and distribution of links, underlies the mechanical properties of the hydrogel samples. Therefore, the network connectivity has been investigated by characterizing the population of links in each sample. First of all, links are defined by localized sets of short contacts (distance < 3.6 Å) among atoms on two separate polysaccharide units, where *units* are nanofibers as well as single cellulose and callose chains. Each link usually involves several pairs of atoms satisfying the distance constraint. In several cases, the number *nc* of short contacts in a single link is up to *nc* ∼ 40, belonging to one or a few consecutive rings on each side. Notice that each atom might participate in more than one pair, therefore the total number of atoms involved usually is less than 2 × *nc*. As it will be apparent from the analysis of hydrogen bonding discussed below, links’ formation is primarily due to dispersion interactions and moderate cellulose and callose hydrophobicity, causing chains to stick together whenever they cross in water. The number of short contacts (*nc* ∼ 40) in each link is such that the link is practically irreversible, although the number and identity of the participating atoms fluctuate somewhat in time. The simple picture of links proposed in this paragraph also presents a few exceptions. In a few cases, for instance, pairs of single chains are coiled on each other along fairly long segments, the link is not well defined, while the number of short contacts may reach a few hundreds. Moreover, as expected, cellulose chains belonging to the crystal fibrils display a large number of short contacts (a few hundreds) among themselves and these extended intrafiber links that do not contribute to the network connectivity are not considered in the present analysis.

**Figure 6:**
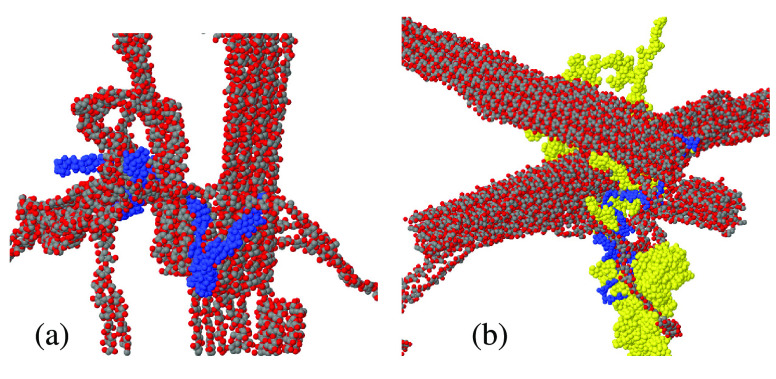
Snapshots showing the network of nanofibers and chains formed within ∼100 ns in the 76:24 cellulose/callose hydrogel sample. (a) Single cellulose chain (blue) joins one nanofiber and several other cellulose single chains. (b) Callose chain (blue) connects cellulose nanofibers, single cellulose chains, and other callose chains (yellow) in the same sample. Cellulose is represented by the dark gray (C) and red (O) dots. All structural units in the two panels are connected to the blue chain (either cellulose or callose) by the short links defined in the text.

Despite the simplicity of the initial configuration and the relatively low number of nanofibers and single chains, the network they form within ∼100 ns is remarkably complex, as can be appreciated by visual analysis of simulation snapshots (see Figure 6). The quantitative analysis shows that, in all (100 – *x*):*x* samples, nanofibers do not form direct links with each other. Moreover, no single chain, either cellulose or callose, is floating alone in water, i.e., without links to other chains or nanofibers. Instead, single chains, either cellulose or callose, are very effective in making multiple links among nanofibers and other single chains. In all the simulated samples, each cellulose chain makes, on average, between 10 and 15 links to other chains, including several links to nanofibers. Because of their reduced extension, callose chains form less links (5–7) per chain, corresponding to about one-half of those originating from cellulose chains. All together, the simulated network appears to be remarkably well connected. Because of the lower number of links involving callose, the connectivity and, presumably, the stability and mechanical properties of the network, decrease somewhat with increasing callose content. These results contrast with the general idea that callose enhances the mechanical properties of polysaccharide mixtures. The disagreement is likely to be due to the fact that polysaccharide mixtures in biological samples are much more structured than the simple, random-like assemblies of simulated hydrogels.

The local, real-space analysis of the hydrogel structure is complemented by the global, Fourier space analysis summarized by the structure factors. The structure factor of water is defined as follows:
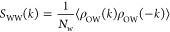
1where *N*_w_ is the number of water molecules in the system, and ρ_OW_(*k*) = ρ_OW_^*^(−*k*) is the Fourier transform of the instantaneous density of the water oxygen atoms (OW), whose position is {**R**_*i*_, *i* = 1, ..., *N*_w_}:
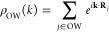
2where, in the simulation, **k** is a reciprocal lattice vector of the real space lattice defined by the pbc and ⟨···⟩ indicates time or ensemble average.

**Figure 7:**
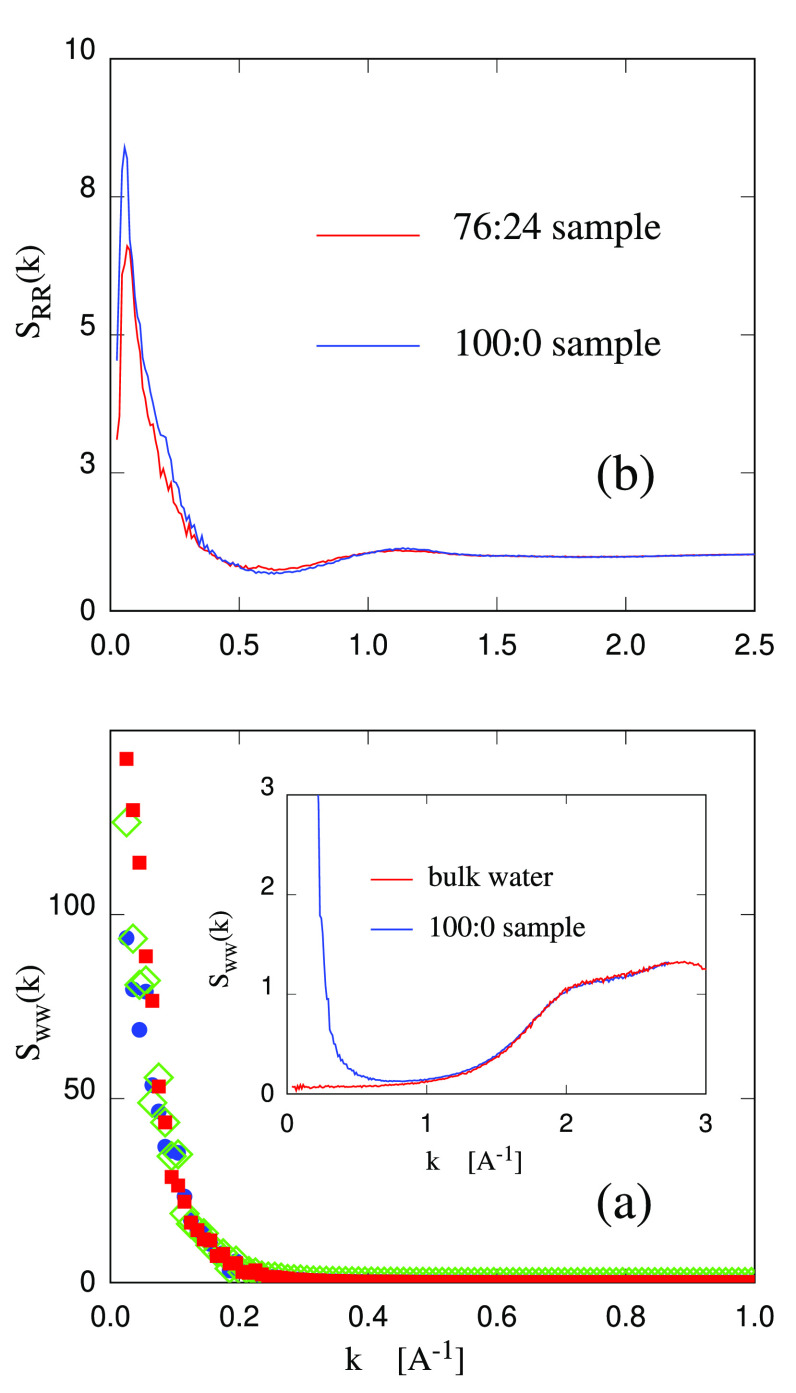
(a) Water structure factor computed according to eq 1. Blue dots, 100:0; green diamonds, 92:8; red squares, 76:24. For the sake of clarity, 84:16 has been omitted. The inset compares the water structure factor computed on the hydrogel 100:0 sample and on bulk water. The difference at *k* ≤ 1 reflects the relative position of polysaccharide nanofibers and chains devoid of water molecules. (b) Structure factor of the polysaccharide fraction, in which each pyranose ring is represented by a single particle located at the geometric center of mass of the ring itself (see section III).

At *k* ≥ 1 Å^–1^, the *S*_WW_(*k*) partial structure factor describes the short-range correlation among water molecules. Since the sample is made primarily of water, in this range *S*_WW_(*k*) is close to the structure factor computed in the same way for a pure homogeneous water sample at the same (*T*, *P*), as shown in the inset of Figure 7a. At low *k* (*k* < 1 Å^–1^), the structure factor of pure water *S*_bulk_(*k*) is monotonic. In particular, in the limit *k* → 0, *S*_bulk_(*k*) is low, i.e., ≪1, because it reflects the low isothermal compressibility of water at ambient conditions.^57^

At variance from the homogeneous water case, the partial structure factor *S*_WW_(*k*) of water defined in eq 1 and computed for the hydrogel samples, displays a prominent peak centered at the origin (*k* ∼ 0.05 Å^–1^, see Figure 7a), due to the presence of mesoscopic features like nanofibers and polymeric chains that modulate the water distribution in space.

The structure factors computed for all the hydrogel samples are the same to within the error bar (see again Figure 7a,b), showing that the replacement of cellulose with callose does not affect the overall structure of the hydrogel very much, as well as its thermodynamic stability (which would affect the *k* ≪ 1 Å^–1^ range). The width of the peak at half-height is Δ*k* = 0.06 Å^–1^, corresponding to a correlation length of about 10 nm, which coincides with the separation of (nearly) parallel crystal nanofibers in the suspension. Apparently, the computed *S*_WW_(*k*) is more sensitive to the regions virtually devoid of water molecules corresponding to the position of the crystalline nanofibers than to the diffuse disturbance due to the isolated chains. However, the background signal between *k* = 0.06 and about *k* = 0.2 Å^–1^ can easily account for correlations in the distribution of single chains, either cellulose or callose.

The partial structure factor of pyranose rings has been computed as well. No distinction is made between cellulose and callose rings. Also, in this case, the result is virtually the same for the samples 100:0, ..., 76:24, and in Figure 7b is reported the result for the two extreme compositions, corresponding to 100:0 and 76:24. In the figure, it is possible to distinguish a broad peak at 1.17 Å^–1^ or 5.4 Å in real space, which is also found in X-ray diffraction data and attributed to the spacing of parallel dense atomic planes in ref (54). The same spacing (5.2 Å, to be precise) also corresponds to the average separation of saccharide rings in covalently connected chains. On the scale used in Figure 7b, to represent the low-*k* prepeak, this peak at 1.17 Å^–1^ is hardly visible, but it would represent the main peak in a similar ring–ring structure factor computed on a homogeneous polysaccharide sample. Even less apparent on the same scale, is a shoulder at *k* ∼ 1.7 Å^–1^, or 3.9 Å in real space, which corresponds again to a peak found in the diffraction patterns of crystalline cellulose^54^ and attributed to the average separation of parallel dense atomic planes. While these correspondences are crucial to assess the validity of the simulation model and analysis, more interesting in the present context is the prominent peak at low-*k*, due to the unequal distribution of nanofibers and chains in the hydrogel. The width at half-maximum of the low-*k* peak is Δ*k* = 0.2 Å^–1^, corresponding to a correlation length of about 3 nm, significantly shorter than the similar mesoscopic correlation length estimated from *S*_WW_(*k*). Judging from the distance and average size of single chains in solution, this shorter length is likely to reflect both the contribution of nanofibers and of the single chains of the amorphous fraction.

### IV.D Computational Analysis of Hydrogen Bonding and Elastic Properties of Cellulose/Callose Hydrogels

The quantitative analysis of hydrogen bonding was performed for the different hydrogel samples under study. The results (summarized in Table 2) show that the incorporation into the hydrogel does not affect much the intra- and interchain H-bonding in the crystalline cellulose nanofibers, as well as the number and distribution of cellulose–water H-bonding briefly mentioned in the case of single nanofibers in water. As expected, single chains, either cellulose and callose, form nearly the same number of HBs with water than those freely floating in 360 × 10^3^ water molecules discussed at the beginning of the Results and Discussion. Again, intrachain H-bonding in callose is quantitatively more important than in cellulose single chains, mainly because they are somewhat more coiled on themselves. Because of the dilute concentration of callose chains, the number of HBs linking callose to cellulose is relatively small. This picture of H-bonding (Table 2) confirms that the structural stability and resilience of the hydrogel network relies primarily on van der Waals interactions and on the moderate hydrophobicity of cellulose and callose chains that favors their sticking at the linkage points discussed in previous paragraphs.

**Table 2:** Average Number of H-Bonds Among the Different Components in the Hydrogel Samples 100:0 to 76:24^a^

sample	100:0	92:8	84:16	76:24
cellulose–cellulose intra	11700	10815	9790	8865
cellulose–cellulose inter	6070	5510	5005	4400
callose–callose intra		630	1300	1940
callose–callose inter		45	330	570
cellulose–callose		580	705	905
cellulose–water	14525	13200	11400	9870
callose–water		2110	4030	6050

a Numbers refer to the whole sample, whose size and composition is given in Table 1. The error bar is of the order of ±5% for each value.

Beyond a short stage in which links are formed, polysaccharide chains display very limited mobility, both because of their size and for the effect of the links themselves, that lock the chains into an extended network. As a result, an estimate of the chains’ diffusion constant is not achievable during the limited simulation time. In each of the hydrogel systems 100:0 to 76:24, the diffusion constant of water is nearly the same to within the error bar (a better comparison, however, is discussed below), and amounts to 80% of the value computed in homogeneous pure water systems modeled by the same force field (see section S6 in the SI for more details on the computation of the water diffusion constant in the 100:0, ..., 76:24 samples).

The 20% decrease observed in hydrogels is probably due mainly to geometric effects related to the decrease of the space available for diffusion, but the formation of water-polysaccharide HBs might give a minor contribute to the reduction. Along the 100:0, ..., 76:24 sequence, the diffusion constant of water decreases slightly but systematically. Although the change (1.5%) between 100:0 (*D*_W_ = 3.75 ± 0.01 cm^2^/s) and 76:24 (*D*_W_ = 3.70 ± 0.01 cm^2^/s) is comparable with the combined error bar, the systematic trend suggests that the effect is real, possibly due to geometric and H-bonding aspects, driven by the replacement of the 10-chain cellulose nanofiber with an equal number of callose chains spread over the sample and forming a higher number of HB.

The equilibrium volume fluctuations introduced to enforce the constant-*P* condition allow the easy evaluation of the simplest mechanical property, i.e., the bulk modulus *B*, through the relation:
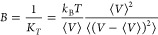
3where *k*_B_ is the Boltzmann constant, and the isothermal compressibility *K*_*T*_ is defined as the reciprocal of *B*.

Since the hydrogels are made primarily by water, their bulk modulus is expected to be relatively close to that of bulk water. Moreover, the connected network of nanofibers and single polysaccharide chains will increase the system rigidity, therefore, the bulk modulus, and, more in general, elastic constants, are expected to be enhanced by the addition of polysaccharides. These expectations are borne out by the simulation results, showing that the bulk modulus of the 100:0 sample (10 wt % cellulose in water) is 10% higher than that of water. Moreover, the bulk modulus of the other hydrogel samples are the same as the one of 100:0 to within the error bar, estimated at about 2% of the bulk-water value. The quoted 2% error bar is only the statistical error. It is possible that systematic errors due to long relaxation processes not adequately sampled by simulation could increase significantly the uncertainty. The inability of simulations to estimate the dependence of *B* on the relative cellulose-callose concentration is likely to be due to the fact that the sought for effect represents a second order variation (due, first, to the mixing of water and polysaccharides and, second, to the replacement of callose to cellulose at equal polysaccharide content) of a quantity (*B*) that is already large in the reference bulk water state.

A more sensitive comparison of experimental and computational results for the hydrogel mechanical properties could be achieved by computing the Young’s modulus (*Y*) of the (100 – *x*):*x* samples, since this same quantity has been measured experimentally^18^ as a function of the relative cellulose–callose concentration. Moreover, since the Young’s modulus of a liquid or suspension is zero (or, better, undefined), the computation of a nonvanishing *Y* is a major diagnostic step to verify the hydrogel state of the simulated samples. On the other hand, the hydrogels under investigations consist of rather dilute networks, and the values of *Y*, determined in ref (18), range between 100 and 200 kPa, that is hardly measurable by simulation. Nevertheless, the estimation of *Y* by simulation has been attempted at first following the fluctuation route, in analogy with the estimation of *B* by eq 3. This approach rigorously provides the linear part of the system response to a uniaxial stress, evaluated (in the present case) at zero stress. To this aim, the constraint of a fluctuating cubic box has been removed, allowing for more general fluctuations. Short preliminary tests have shown that the fully unconstrained dynamics (6 degrees of freedom to describe the simulation box) of the simulation box is too noisy to allow the accurate determination of all the elastic constants within an acceptable simulation time. As a compromise, the simulation cell dynamics has been limited to the fluctuations of the sides (*L*_*x*_, *L*_*y*_, *L*_*z*_) of an orthorhombic box. In this picture, the strain tensor is limited to the three independent diagonal components:
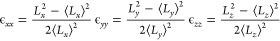
4Assuming that the equilibrium structure has cubic symmetry, the two independent elastic constants can be computed as:
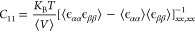
5
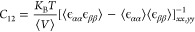
6from which the Young’s modulus *Y* is computed as:
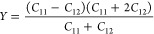
7Needless to say, in the cubic symmetry, statistics can be improved by exploiting the equivalence (*xx*, *xx*) ≡ (*yy*, *yy*) ≡ (*zz*, *zz*) and (*xx*, *yy*) ≡ (*xx*, *zz*) ≡ (*yy*, *zz*). The results of this approach based on averages 70 ns long are reported in Table 3.

**Table 3:** Bulk Modulus *B* (GPa) and Young’s Modulus *Y* (MPa) Estimated from Volume and Shape Fluctuations for the Four Hydrogel Samples 100:0, ..., 76:24^a^

sample	*B* (GPa)	*Y* (MPa)
water	1.75 ± 0.05	
100:0	1.96 ± 0.05	82 ± 3
92:8	1.95 ± 0.05	76 ± 3
84:16	1.99 ± 0.05	71 ± 3
76:24	1.97 ± 0.05	74 ± 3

a The bulk modulus of water has been computed in the same way on a homogeneous sample of 200 × 10^3^ water molecules. The simulation value agrees with previous estimates based on the SPC model, which underestimates the experimental value by about 18% (see ref (58)).

The first and most important observation is that the simulation results greatly overestimate the experimental value. On the positive side, one can observe that on the simulated length and time scales, the sample is solid-like, resisting changes in the shape of the simulation cell. In other terms, despite the dilute character of the polysaccharide network, the system behavior is qualitatively different from a liquid suspension of cellulose and callose in water. A more complete characterization of the hydrogel state would require the computation of frequency-dependent viscoelastic properties, that has not been attempted in the present study. Also positive is the observation that in all cases *Y* is 3 orders of magnitude lower that that of crystalline cellulose measured along the nanocrystal axis, estimated at 130 GPa in refs (54 and 59), comparable to the *Y* = 190 GPa of high quality stainless steel.^60^ The hydrogel value given by simulation is also 1 order of magnitude less than the value measured on low-density polyethylene, implying that the simulated samples are soft on the scale of most common materials. Moreover, the ordering *Y*_100:0_ > *Y*_92:8_ ∼ *Y*_84:16_ ∼ *Y*_76:24_ of the experimental data is qualitatively reproduced, even though quantitative values and the exact ratios are far from the experimental values, as discussed in section V.

To gain insight into the reasons of the observed discrepancy, the Young’s modulus estimation has been repeated using the finite deformation approach, applying an uniaxial stress σ along one of the axes, and measuring the change in the corresponding average side length of the sample. Despite the unfavorable error bar in estimating the effect of applying a tiny axial stress σ, this direct approach gives interesting information. Although the results do not solve the discrepancy between simulation and experiments, they point to subtleties in comparing *Y* computed according to its statistical mechanics definition and measured by AFM (Atomic Force Microscopy) nanoindentation.

**Figure 8:**
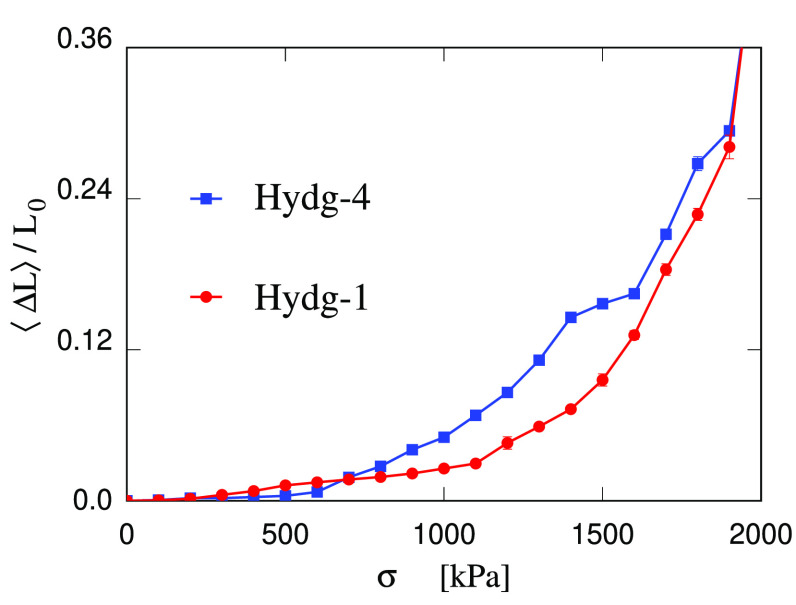
Strain ⟨Δ*L*⟩/*L*_0_ in the hydrogel samples as a function of the applied uniaxial stress σ. Red dots and line: 100:0 sample; blue squares and line: 76:24 sample. The lines are a guide to the eye.

The simulation results for the strain Δ*L*/*L*_0_ as a function of the applied stress σ are reported in Figure 8 for the 100:0 and 76:24 samples. Each point represents the time average over 2 ns. Exploiting the fact that the simulated sample is stiffer than the experimental one, the range of applied σ is also much wider than the one that has been applied in the experiments of ref (18). As a result, the relative statistical error bar is reduced to acceptable levels.

The first observation is that the strain-stress relation is far from linear, and the stiffness of the sample decreases significantly with increasing applied stress σ. In the linear case:
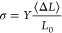
8hence, *Y* is the inverse of the (constant) derivative *d*(⟨Δ*L*⟩/*L*_0_)/d*σ*. To capture nonlinear effects, this last relation is generalized to the real case in which *d*(⟨Δ*L*⟩/*L*_0_)/d*σ* depends on σ. Then, the analytical differentiation of a Padé fit to the computational ⟨Δ*L*⟩/*L*_0_ versus σ relation allows to estimate the σ dependendence of the Young’s modulus. Excluding the lowest-σ points whose computation is the most uncertain, in the case of 100:0 the Young’s modulus is estimated at *Y* = 50 MPa at σ = 0.3 MPa (3 bar), and at *Y* = 4 MPa at σ = 1.5 MPa (15 bar). In the case of 76:24, the estimates are as follows: *Y* = 48 MPa at σ = 0.3 MPa, and *Y* = 4 MPa at σ = 1.5 MPa. In other terms, the estimate of *Y* changes by 1 order of magnitude with σ changing by a few bar, a pressure variation large in macroscopic terms, but very small on the size scale of simulations and of the AFM tip used for the experimental determination. Both samples show clear signs of breaking down at σ ≳ 1.6 MBar, manifested by the rapid increase of ⟨Δ*L*⟩/*L*_0_ with increasing σ. This stability range of the simulated sample is still much wider than the experimental one. However, long-time relaxation processes leading to the progressive elongation of the sample are apparent already around σ = 1 MBar, pointing to a creep-type failing at this reduced stress. It is apparent that in labile and disordered systems like hydrogels, any estimate of mechanical properties, of plasticity and stability limits depend strictly on the time scale underlying the estimation.

The second apparent fact is that, despite the order of magnitude uncertainty in *Y* due to nonlinearity, the simulation estimate is still about 2 orders of magnitude higher than the experimental one. One likely reason of the remaining large *Y* overestimate is the microscopic duration of the simulation, that prevents the sampling of the slow, long-wavelength relaxations/fluctuations in the geometry of the hydrogel. Even the AFM indentation measurements, that cover mesoscopic length scale, last much longer than simulation, giving the hydrogel sufficient time to adapt to the tip progression at nearly the lowest free energy cost. The time scale problem might be enhanced by a purely technical detail, related to the usage of short relaxation times in the constant-pressure algorithm,^31^ that we tried to overcome using a fairly long τ = 50 ps, instead of the 1–2 ps usually adopted in simulation. At this stage the preliminary conclusion is that the estimation by simulation of the elastic properties of very soft materials is challenging since it requires long averaging times for large systems, as well as a careful tuning of all the simulation parameters which determine the volume and shape fluctuations.

### IV.E Models of Dense Cellulose–Callose Structures

**Figure 9:**
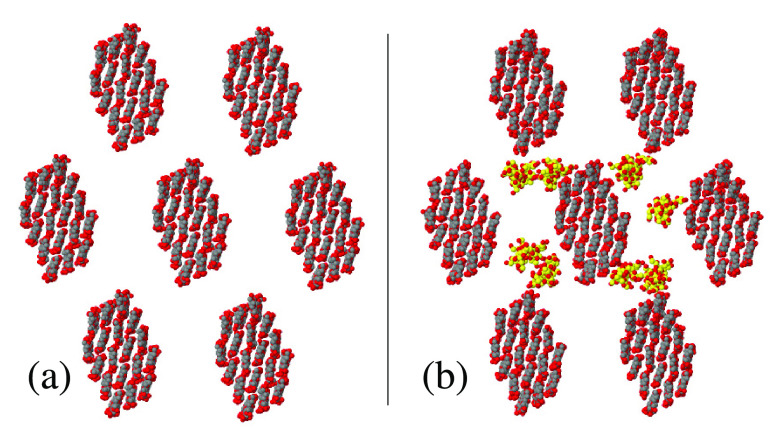
Cross section of the cellulose nanofibers bundle used to investigate compact structure made of cellulose and callose. Panel (a) shows the initial geometry of the seven 18-chain nanofibers representing the cellulose fraction of the sample. Panel (b) shows the bundle at an intermediate stage (8 callose chains) of callose loading. Gray dots: C atoms; red dots: O atoms. H atoms are not shown.

Simulations have been carried out for dense cellulose and callose mixtures to investigate the role of callose in gluing together multiple cellulose nanofibers into bundles, smoothing their contact through surfaces of different quality and orientation. To this aim, seven 18-chain cellulose nanofibers, already equilibrated separately for 100 ns, have been arranged according to the geometry shown in Figure 9a. The nanofibers consist of extended chains, periodically repeated and without terminations. The length of the chains (16 rings) determines the size of the periodic box along the nanofiber axis (about 8.5 nm), while the size of the simulation box in the perpendicular plane is significantly larger (18 nm on each side). The initial arrangement left nanometer spacings among the nanofibers, in which 20 callose chains have been inserted at random position, representing 19 wt % of the sample (see Figure 9b). Also, callose chains extend to infinity through periodic boundary conditions, and their overall orientation is parallel to the cellulose nanofiber axis. Their length, however, is significantly longer (24 d-glucose units, joined by (1,3)-β glucosidic links). In this way, callose chains are partly folded to fit into the simulation cell, and, although they run nearly parallel to the nanofibers axis, they still have some limited possibility of winding around the cellulose nanofibers in the interstitial spaces. The system prepared in this way has been relaxed, giving a solid bundle (labeled Bund-1, see Table 1), and further equilibrated for 100 ns. Considering the solid-like character of the sample, the constraint of periodicity and the long relaxation times of polymeric chains, also in this case the equilibration achieved in 100 ns is limited to local relaxation, but also most real systems are likely to be metastable structures, whose geometry is determined by the biological processes responsible for their formation.

**Figure 10:**
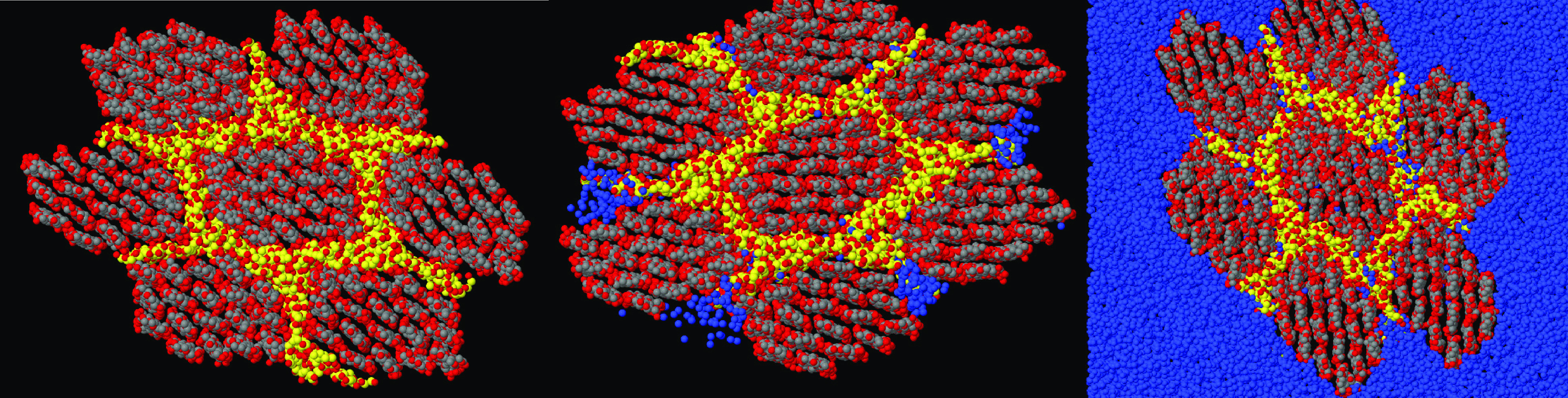
From left to right: cross section of Bund-1, Bund-2, and Bund-3, respectively. The systems composition is summarized in Table 1. The carbon atoms of callose are painted yellow; the oxygen atoms of water are painted blue. The scale of Bund-3 is slightly reduced to show the amount of water in the sample.

**Figure 11:**
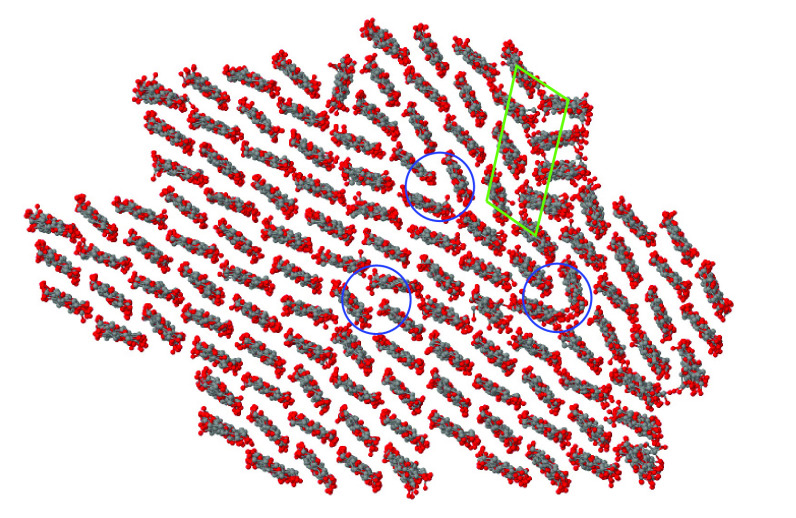
Cross section of a large Iβ nanocrystal made by relaxing the cellulose bundle shown in Figure 9a. The nanocrystal displays dislocations (three of them are highlighted by the blue circles), a grain boundary (highlighted by the green box), and a few point defects.

The result of the relaxation is illustrated in the left panel of Figure 10. The gluing role is apparent by comparing the structure with that of a similar bundle relaxed without the callose chains (see Figure 11). Even with the most careful alignment of the equilibrated (and, thus, slightly disordered chains), joining clean nanofibers in a bundle results in dislocations, grain boundaries, and other extended defects (see Figure 11) that are likely to weaken the strength of the composite structure. The nanofibers in the bundle relaxed with the callose interstitial chains and appear less strained and free of extended defects, with callose smoothing the contact between the nanofibers planar faces, thus, presumably enhancing the resilience of the composite structure (Figure 10).

Water was added to the cellulose–callose system at ∼1 wt % (labeled Bund-2, see Table 1) which then was relaxed for another 100 ns. The simulation shows the progressive and fairly fast penetration of water molecules into the interstitial spaces occupied by callose, reaching well inside the bundle (middle panel of Figure 10). More precisely, water molecules are added at random positions in the simulation cell outside the volume occupied by the bundle. Since at room temperature the vapor pressure is low, within a ns all water molecules are adsorbed on the bundle surface. Water retains a sizable mobility on all cellulose surfaces,^49^ and in a few more ns, all H_2_O molecules migrate to the interstitial spaces, where their mobility is apparently lower, but sufficient to penetrate deep inside the sample. Adding water to the sample without callose results in a similar water adsorption on and successive migration along the surface, with water accumulating into crevices, but never progressing as deep into the bundle as in the callose case (see section S7 in the SI).

As a final step, the full simulation cell measuring 18 × 18 × 8.5 nm has been filled with water using the solvate routine of the Gromacs package, resulting in a sample of 126 cellulose and 20 callose chains, hydrated by 26752 water molecules (labeled Bund-3, see Table 1). The result is similar to the previous one, with water entering the space occupied by callose, making a limited number of HBs with cellulose, and retaining a residual mobility even in proximity of the bundle (right panel of Figure 10).

The cross section of the three bundles that have been simulated at this stage is shown in Figure 10. Simulations have been carried out in the NVT ensemble for the two samples that include empty space around the bundle and in the NPT ensemble for the last sample, in which the simulation box is filled with water. The three systems have been simulated for 150 ns. After ∼50 ns the samples do not show any definite evolution, but fluctuate in time because of their temperature *T* = 300 K. In several aspects, the analysis of their simulation trajectories mainly conforms to the results already discussed for the hydrogel models.

The determination of H-bonding, whose results are summarized in Table 4, shows that both in callose and cellulose intramolecular HBs prevail on intermolecular HBs and confirms the relatively limited role of H-bonding in the overall cohesive energy balance. With increasing callose and water content, the intramolecular H-bonding of cellulose decreases somewhat. Once the different number of d-glucane units belonging to the two species in our samples is taken into account, it is apparent that callose–water H-bonding is somewhat more important than cellulose–water H-bonding. Overall, however, both for cellulose and callose the H-bonding with water is non-negligible, but also not extensive.

**Table 4:** Average Number of HBs Among the Different Components^a^

	sample Bund-1	sample Bund-2	sample Bund-3
cellulose–cellulose intra	931	894	841
cellulose–cellulose inter	507	532	510
callose–callose intra	186	180	173
callose–callose inter	67	59	61
cellulose–callose	143	141	151
cellulose–water		82	640
callose–water		59	210

a Numbers refer to the whole sample, whose size and composition is given in Table 1. The error bar is of the order of ±10 HBs over the total. The cellulose fraction consists of 2016 d-glucose units; the callose fraction consists of 480 d-glucose units.

**Figure 12:**
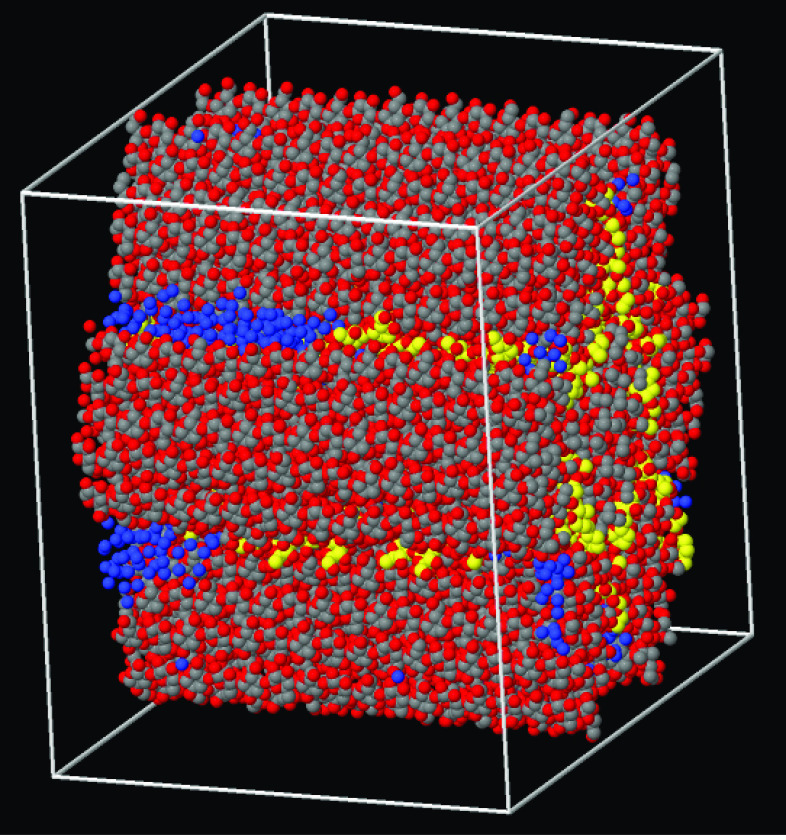
Side view (slightly tilted, as shown by the bounding box) of sample Bund-2, whose surface is contaminated by water.

**Figure 13:**
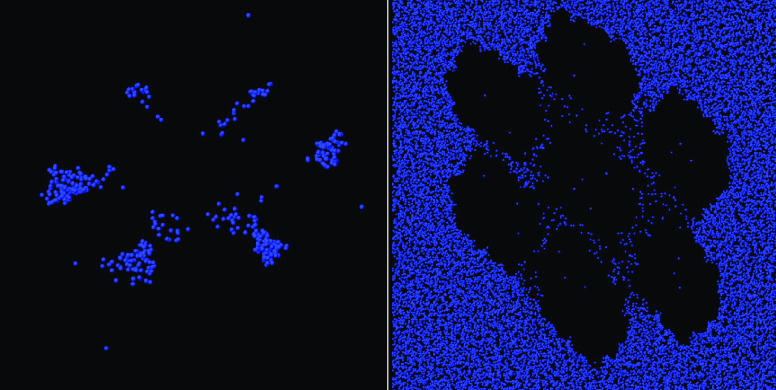
Cross section of samples Bund-2 (left) and Bund-3 (right). Cellulose and callose atoms have been removed to highlight the penetration of water into the bundle, as well as the preference of water for the interstitial callose layer. Only the water oxygen is shown, painted in blue.

More interesting might be the results for water on the surface of the initially dry bundles. The addition of 448 water molecules shows that, despite the expected low affinity of water for cellulose-like polysaccharides, water sticks to the surface of the bundle. The initial uniform distribution of water over the surface relaxes into a patchy distribution (see Figure 12), with water accumulating along the grooves whose bottom is made of callose, with some limited but deep diffusion into callose itself, probably following pores in the callose distribution (see Figure 13, left panel). This drop-like distribution is characteristic of interfaces between immiscible phases, in this case, represented by water on a moderately hydrophobic surface. Being immiscible does not mean that water and callose or even water and cellulose do not attract each other. It only means that, given the choice, water will prefer to interact with water instead of callose and, even more, instead of cellulose. Snapshots of the bundle in bulk water confirms the penetration of water through callose, while water and cellulose are virtually (but not exactly) phase separated. To highlight the penetration of water into the callose range, Figure 13 (right panel) reports only the water oxygen atoms, while cellulose and callose have been removed.

Given the similarity of callose and cellulose, it is likely that the preference of water for callose is due to its amorphous structure, giving origin to local environments more favorable than average for absorption and to pores through which water can penetrate inside. It might be worth pointing out that the enhanced hydration of cellulose structures may affect a number of applications, especially in nanotechnology.^61^

### IV.F Experimental Determination of Callose Effects on Hydrogel Hydration and Dye Loading

**Figure 14:**
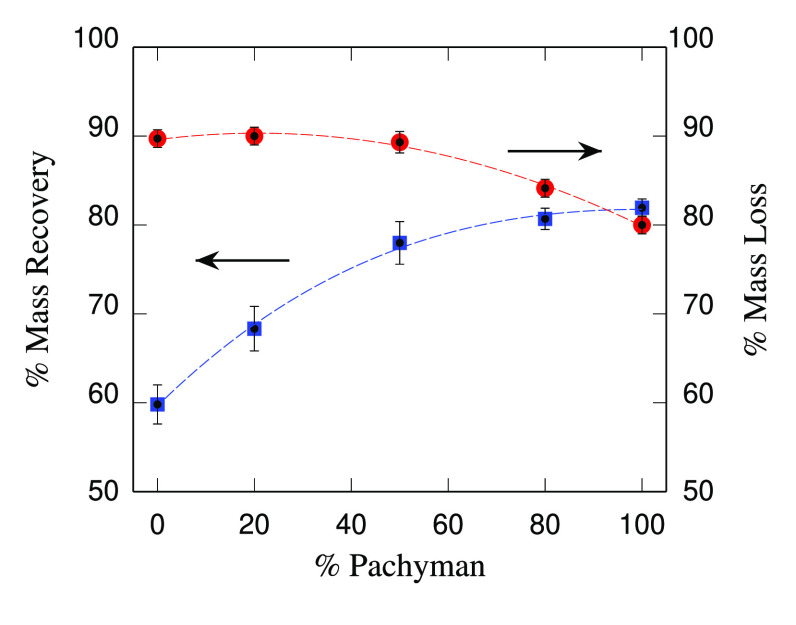
Pachyman increases the water uptake capacity of cellulose. The graphs show mass loss after freeze-drying the hydrogels (brick red line and symbols) and mass recovery after full rehydration (black line and symbols). The *x* axis shows the percentage of Pachyman in each hydrogel. The results suggest an increase in water uptake (i.e., mass recovery) as the Pachyman concentration in the gel increases. The results are from three independent replicas and errors are standard deviation.

Although no quantitative comparison can be made, qualitative mutual support could be recognized between the simulation results and the experimental analysis of water uptake by dried cellulose–callose hydrogels, following the protocol described in section II.C. The graphs in Figure 14 show the % mass loss and % mass recovery versus Pachyman concentration (relative to the total 10 wt % polysaccharide fraction) in the gels.

Increasing callose concentration (% of Pachyman in the gel) increases the % weight recovery after rehydration. The trend is the same in hydrogels containing Curdlan, as can be seen in Figure S12 of the SI. Differences between 100% cellulose and 50% cellulose samples are substantial. In qualitative agreement with the simulation observations, the experimental results suggest that (1,3)-β-glucan has a higher affinity for water (hydrophilicity) than cellulose. Callose might also open nanometric paths for the penetration of water into dense polysaccharide aggregations, thus its addition improves water uptake (rehydration ratio). This observation qualitatively agrees with the results of simulations in the previous subsection.

**Figure 15:**
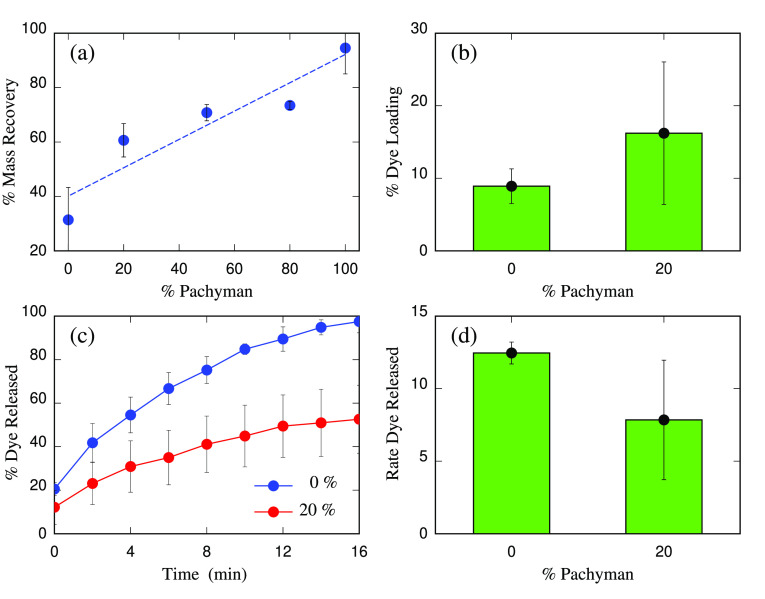
Hydrogels containing Pachyman (commercial (1,3)-β-glucan) display higher dye loading and slower dye release compared to cellulose gels. Graphs show (a) mass recovery after full rehydration of freeze-dried hydrogels used for dye loading/release, (b) percentage of dye loading after 24 h immersion of the gels in methylene blue solution, (c) percentage of dye released measured at 2 min intervals and for a total of 16 min, blue shows 100% cellulose, red shows 20% Pachyman, (d) the rate of the dye released when comparing 20% Pachyman and 100% cellulose hydrogels (0% Pachyman). Data presented in panel (a) are adjusted to a linear trend with a *R^2^* = 0.87. Error bars are standard deviation.

Water hydration properties usually correlate with hydrogel capacity to uptake and release dyes. To further evaluate the structural differences, the hydrogels were loaded in 2 ml of methylene blue solution in water for 24 h. Dye loading in the hydrogel was calculated by determining the dye concentration of the aqueous solution before and after hydrogel loading. Dye molecules successfully diffused into the swollen hydrogels as shown in Figure 15. A higher percentage of dye loading was observed for hydrogels containing 20% of Pachyman when comparing to 100% cellulose. To evaluate the rate of release, the dye loaded gel were submerged in 10 mL of water and the absorbance of the aqueous solution was measured each two minutes for 16 min. The results (Figure 15) show that the rate of dye release is slower in hydrogels containing callose analogues than in 100% cellulose (see also Figures S13 and S14 in the SI for Curdlan). As expected, dye loading/release in the hydrogels was related to the rehydration ratio, callose-containing hydrogels showed higher dye loading/slower release rate than 100% cellulose gels. The differences are likely due to the pores created by (1,3)-β-glucan, maximizing dye loading in the internal aqueous phase of the gel, and slowing down its release. Alternatively, different interactions might exist between cellulose and (1,3)-β-glucan and the dye molecules affecting its release. The rehydration capacity and dye loading/release for the hydrogels show promise in drug delivery applications and expose structural differences predicted in the computational models.

## V Summary and Conclusions

Molecular dynamics simulations based on an atomistic force field have been carried out to investigate structural and mechanical properties of a hydrogel consisting of cellulose and callose in water. The simulated samples are made of about 1.6 × 10^6^ atoms, have a linear size of 25 nm, and, for every composition, the simulation covers a few hundred ns (>300 ns). The computational samples lacks the complexity of natural callose (e.g., the presence of β-1,6-linked branches) but closely mimic hydrogels of the same composition and presumably similar structure recently investigated by experiments.^18^ The aim of the experimental and computational investigations whose results are presented in the previous sections has been to gain insight into specific aspects of the plant cell wall, whose real life complexity prevents a direct approach by atomistic simulations. The task remains challenging because, although incommensurably simpler than a real cell wall, the hydrogel model system is still complex on the scale of current atomistic simulations. The system complexity, in particular, is reflected in the large sizes and long simulation times required to approach the properties of the real hydrogel system. In analogy with general polymer models, the resulting systems are characterized by (i) suitable partial structure factors, whose low-*k* limit reflects the nanostructuring of the hydrogel; (ii) by the computation of the probability distribution of the end-to-end separation of cellulose and callose single chains; (iii) by the estimation of mechanical properties exemplified by the bulk and Young’s modulus of the hydrogel; (iv) by the analysis of the elastic/inelastic transition for the two extreme compositions in cellulose and callose.

Both in experiments and in the present simulations, the sample is made of 90 wt % water, while cellulose and callose account for the remaining 10 wt %. In the simulation samples, the relative proportion of cellulose and callose goes from 100: 0 to 76: 24 wt %. Loosely following experimental information obtained by X-ray diffraction, in each sample 56% of cellulose is present as crystalline nanofibers, each consisting of 10 cellulose chains. The remaining 44% of cellulose, as well as the totality of callose, represents an amorphous polysaccharide fraction, introduced into the sample as single chains of variable orientation. Within ∼100 ns, the evolution of chains gives origin to a multitude of links that connect the crystalline nanofibers into an elastic network. Hydrogen bonding contributes somewhat to the linking of different units, but it is apparent that dispersion (van der Waals) interactions play the major role, causing the close contact of several ion pairs for every individual link. The picture emerging from simulation of an assembly of cellulose crystalline nanofibers linked together by floating cellulose and callose chains is closely reminiscent of the one proposed in ref (62) (see also ref (34)) for the plant cell wall. In the original formulation, the tethers stretching from one nanofiber to the neighboring ones were xyloglucan chains. In a revised version, the links were made of pectin.^63,64^ Our experiments and simulations suggest that callose could fill the tethering role, especially when enhanced resilience is required to face challenges to the cell wall integrity.

The first result of the quantitative analysis is that the simple procedure outlined in the previous sections gives origin to cellulose, callose and water mixtures that indeed represent hydrogels, since the samples display stability, homogeneity, connectivity and elastic properties beyond those of a liquid suspension of polysaccharides in water. Moreover, analysis of the structure and bonding shows that the relatively uniform distribution of the saccharide nanofibers and chains is not due to the solubility of cellulose and callose, but to the formation of a stable network through a number of cross-links among the various sample components. From the computational point of view, this is already a nontrivial observation, since hydrogels are paradigmatic complex systems, whose investigation by atomistic MD has only recently become feasible.^65−67^

Besides the qualitative agreements between simulation and experiments, quantitative disagreements are also apparent, especially in the estimation of mechanical properties such as the bulk modulus *B* and the Young’s modulus *Y*. These quantities, computed from fluctuations of the volume and shape of the simulation cells, are significantly overestimated by simulation (see Table 3). Hydrogel properties, in particular, depend on the choice of the molecular building blocks, and on the network topology of the hydrogel, which, in turn, depends on the preparation history. The agreement of computational and experimental values for the properties of hydrogels, therefore, relies on the quality of the molecular force field, and on the ability to reproduce the supramolecular organization of the sample. Despite known limitations, the ability of popular force fields to model cellulose and callose chains is generally considered at least sufficient. The comparison of mechanical properties, therefore, is primarily a test of the cellulose and callose supramolecular structure. The observed overestimation of the Young’s modulus, therefore, says that the procedure adopted to prepare the computational sample is too simple, and the relaxation stage probably too short. As a result, the distribution of crystalline nanofibers and single chains is too ordered, possibly because the chains are introduced in the sample as extended, and almost immediately start to form links with nanofibers and other molecules, before folding into a nearly Gaussian coil. In this way the connectivity of the network is somewhat overestimated, presumably leading to an overestimate of *Y*. Improving this aspect will require further coordinated experimental and computational effort.

For the time being, the simulation results point to important nonlinearity in the stress–strain relation already at low σ. This effect can be explained by the variety of links joining the nanofibers and chains in the hydrogel. Because of disorder and thermal fluctuations, the strength of the links covers a broad distribution, from strong down to very weak. The weak links, in particular, are modified or broken by the application of even modest axial loads, changing the system irreversibly and resulting in the nonlinear response measured by *Y*. This nonlinearity, in turn, might blur the comparison of experimental and computational data. The estimation of *Y* by AFM, for instance, is based on a surface indentation of nonvanishing amplitude, thus mixing linear and nonlinear response coefficients. According to simulations, the nonlinear terms decrease the stiffness of the sample, and any measurement based on a finite deformation will underestimate the linear coefficient *Y*. This argument is supported by the observation that the estimate of *Y* for the cell wall of a pollen tube (also made of a polysaccharide network) carried out by a different experimental technique gives an estimate of 350 MPa.^68^ Admittedly, the system composition and structure are different from those of the hydrogels, but the large difference in the experimental values suggests that the measurement approach can greatly influence the estimated value, and the quantitative comparison with simulation requires consideration of a variety of effects.

One aspect worth emphasizing on the comparison of experimental and computational estimates of the Young modulus (and of any static mechanical property) concerns the time scale of the two measurements. Experimental data on the complex modulus G*(ω) of the same systems investigated in the present study (Figure 4 in the Supporting Information of ref (18).) show that, besides the dependence on the cellulose-callose relative concentration, G*(ω) has an essential frequency dependence, growing by nearly 3 orders of magnitude on the limited frequency range 0.1–100 Hz. At nonvanishing frequency, obtaining *Y* from the complex G*(ω) requires more data than currently available. Nevertheless, the two quantities are closely related. The results obtained by simulations spanning times between the 100 ns and the μs cannot be attributed to a specific frequency, but certainly are affected by the high frequency limit of the mechanical properties, contributing to explain the overestimate of *Y* by simulation. Of course, this observation points to the need of introducing methods to estimate mechanical properties better than plain MD. This could possibly be achieved by free energy methods and accelerated sampling approaches.^69^

Besides the hydrogel simulations, a few other samples have been considered. In particular, simulations have been carried out on samples representing dense cellulose and callose aggregates at various degrees of water contamination, up to full hydration. The results highlight the role of callose in smoothing the contact surface of different nanofibers in forming larger bundles. The results also show that the inherent disordered character of callose opens pores to the penetration of water deep inside nanofiber bundles in which the interfaces are glued by callose. Both observations might play a role in explaining the effect of callose on the mechanical properties and hydration state of cellulose-callose structures. Experimental determination of mass recovery after dehydration and rehydration of the hydrogel and dye loading/release rates support a role for (1,3)-β glucans (i.e., callose) in improving water/dye uptake and slowing down dye release. These physical properties of callose can be connected to its mechanical properties and can be exploited in the design of drug scaffolds.

A complementary outcome of the present investigation is a library of trajectories for systems of variable cellulose and callose concentration in water (at fixed water content) covering a few hundred ns. This database is suitable for tuning a coarse grained model (for instance of the Martini family)^70^ that is needed to extend significantly the size and time scales of the simulation. Equilibrated polysaccharide geometries in the *gro* format suitable for Gromacs are given in the files 100–0.xyz, 92–8.xyz, 84–16.xyz, and 76–24.xyz in the SI. The name of the files corresponds to the cellulose/callose relative concentration in wt %. Complementary data on cellulose–callose hydrogels at 76–23 wt % relative composition contaminated by [emim][OAc] ions are discussed in section S11 of the SI.

The computations described in this paper have been running on CPUs and GPUs of different supercomputers, with a (relatively modest) effort corresponding to a few million (equivalent) core hours. The conversion of CPU and GPU time has been made considering the speed-up of representative runs for the same sample. Using coarse grained and multiscale approaches, a comparable effort could cover significantly larger samples and longer times, allowing for more realistic preparation and more extensive relaxation of samples, hopefully improving the agreement of computed and experimentally measured properties.

On the atomistic simulation side, the next step will be the introduction of more polysaccharide types such as xyloglucan and xylan and possibly peptides (forming, for instance, structures similar to peptidoglycans), since the plant cell wall includes a wide variety of components whose relative concentration changes according to the different stages of cell life. Understanding these processes would open the way to a wide number of new developments in biology and biomedicine, pharmaceutical sciences, and nanoengineering.

## Data Availability

Gromacs input files for all samples (including equilibrated water) are available on request from the authors. Because of size, simulation trajectories are available on reasonable request from the authors. All data produced by the analysis of trajectories and reported in the figures and tables are available as part of the Article. Experimental raw data will also be available on request from the authors.

## Supporting Information Available

Calibration curves for X-ray analysis and dye loading/release, as well as additional rehydration and dye uptake data; Results from preliminary simulations of cellulose crystal nanofibers and crystal surfaces; Data on water adsorption on crystal cellulose bundles and water diffusion in hydrated systems (PDF)
100:0 relative cellulose–callose concentration in wt % (XYZ)
92:8 relative cellulose-callose concentration in wt % (XYZ)
84:16 relative cellulose-callose concentration in wt % (XYZ)
76:24 relative cellulose-callose concentration in wt % (XYZ)

## Supplementary Material

bm3c01396_si_001.pdf

bm3c01396_si_002.xyz

bm3c01396_si_003.xyz

bm3c01396_si_004.xyz

bm3c01396_si_005.xyz

## References

[ref1] Houston K.; Tucker M. R.; Chowdhury J.; Shirley N.; Little A. The plant cell wall: A complex and dynamic structure as revealed by the responses of genes under stress conditions. Front. Plant Sci. 2016, 7, 984 10.3389/fpls.2016.00984. 27559336 PMC4978735

[ref2] Somerville C.; Bauer S.; Brininstool G.; Facette M.; Hamann T.; Milne J.; Osborne E.; Paredez A.; Persson S.; Raab T.; et al. Towards a systems approach to understanding plant cell walls. Science 2004, 306, 2206–2211. 10.1126/science.1102765. 15618507

[ref3] Sobhanadhas L. S. S.; Kesavan L.; Fardim P. Topochemical engineering of cellulose-based functional materials. Langmuir 2018, 34, 9857–9878. 10.1021/acs.langmuir.7b04379. 29694048 PMC6151662

[ref4] Eichhorn S. J.; Rahatekar S. S.; Vignolini S.; Windle A. H. New horizons for cellulose nanotechnology. Phil. Trans. R. Soc. A 2018, 376, 20170200 10.1098/rsta.2017.0200. 29277748 PMC5746563

[ref5] Hasan N.; Rahman L.; Kim S.-H.; Cao J.; Arjuna A.; Lallo S.; Jhun B. H.; Yoo J.-W. Recent advances of nanocellulose indrug delivery systems. J. Pharm. Investig. 2020, 50, 553–572. 10.1007/s40005-020-00499-4.

[ref6] Wang X.; Yao C.; Wang F.; Li Z. Cellulose-based nanomaterials for energy applications. Small 2017, 13, 1702240 10.1002/smll.201702240. PMC5837049 28902985

[ref7] Zhu W.; Droguet B.; Shen Q.; Zhang Y.; Parton T. G.; Shan X.; Parker R. M.; De Volder M. F. L.; Deng T.; Vignolini S.; et al. Structurally colored radiative cooling cellulosic films. Adv. Sci. 2022, 9, 2202061 10.1002/advs.202202061. PMC9475522 35843893

[ref8] Wang B.; Andargie M.; Fang R. The function and biosynthesis of callose in high plants. Heliyon 2022, 8, e09248 10.1016/j.heliyon.2022.e09248. 35399384 PMC8991245

[ref9] Levy A.; Erlanger M.; Rosenthal M.; Epel B. L. A plasmodesmata-associated *β*-1,3-glucanase in Arabidopsis. Plant J. 2007, 49, 669–682. 10.1111/j.1365-313X.2006.02986.x. 17270015

[ref10] Han X.; Huang L.-J.; Feng D.; Jiang W.; Miu W.; Li N. Plasmodesmata-related structural and functional proteins: The long sought-after secrets of a cytoplasmic channel in plant cell walls. Int. J. Mol. Sci. 2019, 20, 2946 10.3390/ijms20122946. 31212892 PMC6627144

[ref11] Radja A.; Horsley E. M.; Lavrentovich M. O.; Sweeney A. M. Pollen cell wall patterns form from modulated phases. Cell 2019, 176, 856–868. 10.1016/j.cell.2019.01.014. 30735635

[ref12] Chen X.-Y.; Liu L.; Lee E.; Han X.; Rim Y.; Chu H.; Kim S.-W.; Sack F.; Kim J.-Y. The Arabidopsis callose synthase gene GSL8 is required for cytokinesis and cell patterning. Plant Physiol. 2009, 150, 105–113. 10.1104/pp.108.133918. 19286936 PMC2675722

[ref13] Verma D. P. S.; Hong Z. Plant callose synthase complexes. Plant Mol. Biol. 2001, 47, 693–701. 10.1023/A:1013679111111. 11785931

[ref14] Luna E.; Pastor P.; Robert J.; Flors V.; Mauch-Mani B.; Ton J. Callose deposition: a multifaceted plant defense response. Mol. Plant-Microbe Interact. 2011, 24, 183–193. 10.1094/MPMI-07-10-0149. 20955078

[ref15] Eggert D.; Naumann M.; Reimer R.; Voigt C. Nanoscale glucan polymer network causes pathogen resistance. Sci. Rep. 2014, 4, 4159 10.1038/srep04159. 24561766 PMC3932449

[ref16] Deslandes Y.; Marchessault R. H.; Sarko A. Triple-helical structure of (1–3)-β-d-Glucan. Macromoles 1980, 13, 1466–1473. 10.1021/ma60078a020.

[ref17] Zhang H.; Nishina K.; Foster T. J.; Williams M. A. K.; Norton I. T. A study of the gelation of the polysaccharide Curdlan. In Stud. Surf. Sci. Catal.; Iwasawa Y., Oyama N., Kunieda H., Eds.; Elsevier Science B.V, 2018; Vol. 132.

[ref18] Abou-Saleh R. H.; Hernandez-Gomez M. C.; Amsbury S.; Paniagua C.; Bourdon M.; Miyashima S.; Helariutta Y.; Fuller M.; Budtova T.; Connell S. D.; et al. Interactions between callose and cellulose revealed through the analysis of biopolymer mixtures. Nat. Commun. 2018, 9, 4538 10.1038/s41467-018-06820-y. 30382102 PMC6208431

[ref19] Rongpipi S.; Ye D.; Gomez E. D.; Gomez E. W. Progress and opportunities in the characterisation of cellulose - Animportant regulator of cell wall growth and mechanics. Front. Plant Sci. 2019, 9, 1894 10.3389/fpls.2018.01894. 30881371 PMC6405478

[ref20] Ciolacu D.; Kovac J.; Kokol V. The effect of the cellulose-binding domain from clostridium cellulovorans on the supramolecular structure of cellulose fibers. Carbohyd. Res. 2010, 345, 621–630. 10.1016/j.carres.2009.12.023. 20122684

[ref21] Shen X.; Shamshina J. L.; Berton P.; Bandomir J.; Wang H.; Gurau G.; Rogers R. D. Comparison of hydrogels prepared with ionic-liquid-isolated vs commercial chitin and cellulose. ACS Sustainable Chem. Eng. 2016, 4, 471–480. 10.1021/acssuschemeng.5b01400.

[ref22] Cornell W. D.; Cieplak P.; Bayly C. I.; Gould I. R.; Merz K. M. Jr; Ferguson D. M.; Spellmeyer D. C.; Fox T.; Caldwell J. W.; Kollman P. A. A second generation force field for the simulation of proteins, nucleic acids, and organic molecules. J. Am. Chem. Soc. 1995, 117, 5179–5197. 10.1021/ja00124a002.

[ref23] Oostenbrink C.; Villa A.; Mark A. E.; van Gunsteren W. F. A biomolecular force field based on the free enthalpy ofhydration and solvation: the GROMOS force-field parameter sets 53A5 and 53A6. J. Comput. Chem. 2004, 25, 1656–1676. 10.1002/jcc.20090. 15264259

[ref24] Schmid N.; Eichenberger A. P.; Choutko A.; Riniker S.; Winger M.; Mark A. E.; van Gunsteren W. F. Definition and testing of the GROMOS force-field versions 54A7 and 54B7. Eur. Biophys. J. 2011, 40, 843–856. 10.1007/s00249-011-0700-9. 21533652

[ref25] Berendsen H. J. C.; Postma J. P. M.; van Gunsteren W. F.; Hermans J. In Intermolecular Forces; Pullman B., Ed.; D. Reidel Publishing Company: Dordrecht, The Netherlands, 1981; pp 331–342.

[ref26] Malde A. K.; Zuo L.; Breeze M.; Stroet M.; Poger D.; Nair P. C.; Oostenbrink C.; Mark A. E. An automated force fieldtopology builder (ATB) and repository: version 1.0. J. Chem. Theory Comput. 2011, 7, 4026–4037. 10.1021/ct200196m. 26598349

[ref27] Darden T.; York D.; Pedersen L. Particle mesh Ewald: an Nlog(N) method for Ewald sums in large systems. J. Chem. Phys. 1993, 98, 10089 10.1063/1.464397.

[ref28] Nosé S. A molecular dynamics method for simulations in the canonical ensemble. Mol. Phys. 1984, 52, 255–268. 10.1080/00268978400101201.

[ref29] Hoover W. G. Canonical dynamics: Equilibrium phase-space distributions. Phys. Rev. A. 1985, 31, 1695–1697. 10.1103/PhysRevA.31.1695. 9895674

[ref30] Parrinello M.; Rahman A. Polymorphic transitions in single crystals: A new molecular dynamics method. J. Appl. Phys. 1981, 52, 7182–7190. 10.1063/1.328693.

[ref31] Berendsen H. J. C.; van der Spoel D.; van Drunen R. GROMACS: A message-passing parallel molecular-dynamics implementation. Comput. Phys. Commun. 1995, 91, 43–56. 10.1016/0010-4655(95)00042-E.

[ref32] Moon R. J.; Martini A.; Nairn J.; Simonsen J.; Youngblood J. Cellulose nanomaterials review: structure, properties andnanocomposites. Chem. Soc. Rev. 2011, 40, 3941–3994. 10.1039/c0cs00108b. 21566801

[ref33] Gomes T. C. F.; Skaf M. S. Cellulose-builder: A toolkit for building crystalline structures of cellulose. J. Comput. Chem. 2012, 33, 1338–1346. 10.1002/jcc.22959. 22419406

[ref34] Cosgrove D. J. Re-constructing our models of cellulose and primary cell wall assembly. Curr. Opin. Plant Biol. 2014, 22, 122–131. 10.1016/j.pbi.2014.11.001. 25460077 PMC4293254

[ref35] Thomas L. H.; Forsyth V. T.; Sturcova A.; Kennedy C. J.; May R. P.; Altaner C. M.; Apperley D. C.; Wess T. J.; Jarvis M. C. Structure of cellulose microfibrils in primary cell walls from Collenchyma. Plant Physiol. 2012, 161, 465–476. 10.1104/pp.112.206359. 23175754 PMC3532275

[ref36] Daicho K.; Saito T.; Fujisawa S.; Isogai A. Crystallinity of nanocellulose: Dispersion-induced disordering at the grainboundary in biologically structured cellulose. ACS Appl. Nano Mater. 2018, 1, 5774–5785. 10.1021/acsanm.8b01438.

[ref37] Kubicki J. D.; Yang H.; Sawada D.; O’Neill H.; Oehme D.; Cosgrove D. The shape of native plant cellulose microfibrils. Sci. Rep. 2018, 8, 13893 10.1038/s41598-018-32211-w. 30228280 PMC6143632

[ref38] Song B.; Zhao S.; Shen W.; Collings C.; Ding S.-Y. Direct measurement of plant cellulose microfibril and bundles in native cell walls. Front. Plant Sci. 2020, 11, 479 10.3389/fpls.2020.00479. 32391038 PMC7193091

[ref39] Rosén T.; He H. R.; Wang R.; Zhan C.; Chodankar S.; Fall A.; Aulin C.; Larsson P. T.; Lindström T.; Hsiao B. S. Cross-sections of nanocellulose from wood analysed by quantized polydispersivity of elementary microfibrils. ACS Nano 2020, 14, 16743–16754. 10.1021/acsnano.0c04570. 33253525 PMC8926302

[ref40] Wu X.; Moon R. J.; Martini A. Calculation of single chain cellulose elasticity using fully atomistic modeling. TAPPI J. 2011, 10, 37–42. 10.32964/TJ10.4.37.

[ref41] Tashiro K.; Kobayashi M. Theoretical evaluation of three-dimensional elastic constants of native and regenerated celluloses: role of hydrogen bonds. Polymer 1991, 32, 1516–1526. 10.1016/0032-3861(91)90435-L.

[ref42] Tanaka F.; Fukui N. Molecular motion of an isolated single chain cellulose molecules. Sen’i Gakkaishi 2004, 60, 261–265. 10.2115/fiber.60.261.

[ref43] Strobl G. The Physics of Polymers; Springer: Heidelberg, 2007.

[ref44] Okobira T.; Miyoshi K.; Uezu K.; Sakurai K.; Shinkai S. Molecular dynamics studies of side chain effects on the β-1,3-d-glucan triple helix in aqueous solution. Biomacromolecules 2008, 9, 783–788. 10.1021/bm700511d. 18257529

[ref45] Feng X.; Li F.; Ding M.; Zhang R.; Shi T.; Lu Y.; Jiang W. Molecular dynamics simulation: Study on the recognitionmechanism of linear β-(1,3)-d-glucan by Dectin-1. Carbohydr. Polym. 2022, 286, 119276 10.1016/j.carbpol.2022.119276. 35337502

[ref46] Wang Y.; Yue T.; Yuan Y. Exploration of binding interaction of *β*-1,3-D-glucan and patulin by molecular dynamics simulation study. J. Comput. Biophys. Chem. 2022, 21, 683–694. 10.1142/S2737416522500284.

[ref47] Wohlert M.; Benselfelt T.; Wagberg L.; Furó I.; Berglund L. A.; Wohlert J. Cellulose and the role of hydrogen bonds: not in charge of everything. Cellulose 2022, 29, 1–23. 10.1007/s10570-021-04325-4.

[ref48] Matthews J. F.; Skopec C. E.; Mason P. E.; Zuccato P.; Torget R. W.; Sugiyama J.; Himmel M. E.; Brady J. W. Computer simulation studies of microcrystalline cellulose Iβ. Carbohydr. Res. 2006, 341, 138–152. 10.1016/j.carres.2005.09.028. 16297893

[ref49] Fularz A.; Rice J. H.; Ballone P. Morphology of nanometric overlayers made of porphyrin-type molecules physisorbed oncellulose I*β* crystals and nanocrystals. J. Phys. Chem. B 2021, 125, 11432–11443. 10.1021/acs.jpcb.1c07261. 34634911 PMC8543442

[ref50] Tang S. K.; Baker G. A.; Ravula S.; Jones J. E.; Zhao H. Peg-functionalized ionic liquids for cellulose dissolution and saccharification. Green Chem. 2012, 14, 2922–2932. 10.1039/c2gc35631g.

[ref51] Park S.; Baker J. O.; Himmel M. E.; Parilla P. A.; Johnson D. K. Cellulose crystallinity index: Measurement techniques andtheir impact on interpreting cellulase performance. Biotechnol. Biofuels 2010, 3, 10 10.1186/1754-6834-3-10. 20497524 PMC2890632

[ref52] Kawashita M.; Nakao M.; Minoda M.; Kim H.-M.; Beppu T.; Miyamoto T.; Kokubo T.; Nakamura T. Apatite-forming ability of carboxyl group-containing polymer gels in a simulated body fluid. Biomaterials 2003, 24, 2477–2484. 10.1016/S0142-9612(03)00050-4. 12695074

[ref53] Ciolacu D.; Ciolacu F.; Popa V. I. Amorphous cellulose - structure and characterization. Cell. Chem. Technol. 2011, 45, 13–21.

[ref54] Nishiyama Y. Structure and properties of the cellulose microfibril. J. Wood. Sci. 2009, 55, 241–249. 10.1007/s10086-009-1029-1.

[ref55] Jarvis M. C. Structure of native cellulose microfibrils, the starting point for nanocellulose manufacture. Philos. Trans. R. Soc. A 2018, 376, 20170045 10.1098/rsta.2017.0045. 29277742

[ref56] Lopez-Sanchez P.; Martinez-Sanz M.; Bonilla M. R.; Wang D.; Gilbert E. P.; Stokes J. R.; Gidley M. J. Cellulose-pectin composite hydrogels: Intermolecular interactions and material properties depend on order of assembly. Carbohydr. Polym. 2017, 162, 71–81. 10.1016/j.carbpol.2017.01.049. 28224897

[ref57] Frenkel D.; Smit B. Understanding Molecular Simulation, 2nd ed.; Academic Press: San Diego, 2002.

[ref58] Jorgensen W. L.; Jenson C. Temperature dependence of TIP3P, SPC, and TIP4P water from NPT Monte Carlo simulations: Seekingtemperatures of maximum density. J. Comput. Chem. 1998, 19, 1179–1186. 10.1002/(SICI)1096-987X(19980730)19:10<1179::AID-JCC6>3.0.CO;2-J.

[ref59] Nishino T.; Takano K.; Nakamae K. Elastic modulus of the crystalline regions of cellulose polymorphs. J. Polym.Sci. B: Polym. Phys. 1995, 33, 1647–1651. 10.1002/polb.1995.090331110.

[ref60] Grujicic M.; Zhao H. Optimization of 316 stainless steel/alumina functionally graded material for reduction of damage induced by thermal residual stresses. Mater. Sci. Eng.: A 1998, 252, 117–132. 10.1016/S0921-5093(98)00618-2.

[ref61] Penttilä P. A.; Paajanen A.; Ketoja J. A. Combining scattering analysis and atomistic simulation of wood-water interactions. Carbohydr. Polym. 2021, 251, 117064 10.1016/j.carbpol.2020.117064. 33142616

[ref62] Hayashi T. Xyloglucans in the primary cell wall. Annu. Rev. Plant Physiol. Plant Mol. Biol. 1989, 40, 139–168. 10.1146/annurev.pp.40.060189.001035.

[ref63] Wang T.; Zabotina O.; Hong M. Pectin-cellulose interactions in the Arabidopsis primary cell wall from two-dimensional magic-angle-spinning solid-state nuclear magnetic resonance. Biochemistry 2012, 51, 9846–9856. 10.1021/bi3015532. 23167456

[ref64] Peaucelle A.; Braybrook S.; Hofte H. Cell wall mechanics and growth control in plants: the role of pectins revisited. Front. Plant Sci. 2012, 3, 121 10.3389/fpls.2012.00121. 22685449 PMC3368173

[ref65] An M.; Demir B.; Wan X.; Meng H.; Yang N.; Walsh T. R. Predictions of thermo-mechanical properties of cross-linked polyacrylamide hydrogels using molecular simulations. Adv. Theory Simul. 2019, 2, 1800153 10.1002/adts.201800153.

[ref66] Kumar R.; Parashar A. Atomistic simulations of pristine and nanoparticle reinforced hydrogels: A review. WIREs Comput. Mol. Sci. 2023, 13, e1655 10.1002/wcms.1655.

[ref67] Koochaki A.; Shahgholi M.; Sajadi S. M.; Babadi E.; Inc M. Investigation of the mechanical stability of polyethylene glycol hydrogel reinforced with cellulose nanofibrils for wound healing: Molecular dynamics simulation. Eng. Anal. Bound. Elem. 2023, 151, 1–7. 10.1016/j.enganabound.2023.02.055.

[ref68] Nezhad A. S.; Naghavi M.; Packirisamy M.; Bhat R.; Geitmann A. Quantification of the Young’s modulus of the primary plant cell wall using Bending-Lab-On-Chip (BLOC). Lab Chip 2013, 13, 2599–2608. 10.1039/c3lc00012e. 23571308

[ref69] Decherchi S.; Cavalli A. Thermodynamics and kinetics of drug-target binding by molecular simulation. Chem. Rev. 2020, 120, 12788–12833. 10.1021/acs.chemrev.0c00534. 33006893 PMC8011912

[ref70] Grünewald F.; Punt M. H.; Jefferys E. E.; Vainikka P. A.; König M.; Virtanen V.; Meyer T. A.; Pezeshkian W.; Gormley A. J.; Karonen M.; et al. Martini 3 coarse-grained force field for carbohydrates. Chem. Theory Comput. 2022, 18, 7555–7569. 10.1021/acs.jctc.2c00757. PMC9753587 36342474

