# Supplementary material for: Cellulose–Callose Hydrogels: Computational Exploration of Their Nanostructure and Mechanical Properties
Source: Biomacromolecules. 2024 Feb 27;25(3):1989–2006. doi: 10.1021/acs.biomac.3c01396 (PMC10934845; doi:10.1021/acs.biomac.3c01396)
Supplement: Supplementary file 1 — bm3c01396_si_001.pdf [file bm3c01396_si_001.pdf]

Supporting information on:

Cellulose-Callose Hydrogels: a Computational Exploration of their Structure and Mechanical Properties

Pallavi Kumari(1,2,3), Pietro Ballone(4,5), Candelas Paniagua(1,6),

Radwa H. Abou-Saleh(2,7,8), Yoselin Benitez-Alfonso (1)*

(1) The Astbury Centre and the Centre for Plant Science,

School of Biology, University of Leeds, Leeds, LS2 9JT, UK

(2) School of Physics and Astronomy, University of Leeds,

Woodhouse Lane, Leeds, LS2 9JT, UK

(3) Present address: Institut für Röntgenphysik (IRP),

Georg-August-Universität Göttingen, Göttingen, Germany

(4) School of Physics, University College, Dublin, Ireland

(5) Conway Institute for Biomolecular and Biomedical

Research, University College, Dublin, Ireland.

(6) Instituto de Hortofruticultura Subtropical y Mediterránea

(IHSM-UMA-CSIC). Dpto. Botánica y Fisiología Vegetal,

Universidad de Málaga, 29071, Málaga, Spain.

(7) Department of physics, Faculty of science, Galala university, Egypt. and

(8) Department of Physics, Faculty of science, Mansoura University, Egypt.

I. CALIBRATION CURVE FOR THE DYE LOAD AND RELEASE

Standard curve

Concentration Methylene Blue (ug/ml)	Absorbance 665nm
**5**	0.926
**2.5**	0.457
**1.25**	0.214
**0.625**	0.093
**0.3125**	0.027

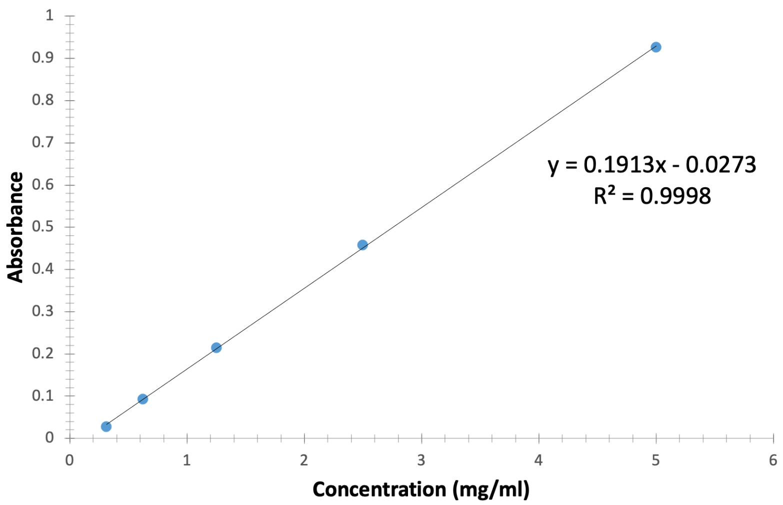

FIG. S1: Calibration curve to assess the dye concentration during loading and release in polysaccharide hydrogels. The curve was obtained as follows: Methylene blue was dissolved in water at a concentration of 250 μg/ml. Several dilutions were prepared and absorbance was measured in a UV-Visible spectrophotometer at 665 nm.

II. COHESIVE ENERGY OF CELLULOSE NANOFIBRES

In addition to a real-time trajectory, the simulation output provides a time dependent and an average potential energy for the simulated sample. The cohesive energy U_{coh} of each cellulose crystalline nanofibre with respect to its constitutive single chains has been computed according to the usual definition:

$$U_{coh}[n_c] = -\left\{\langle U[nanofibre]\rangle - n_c \langle U[single\ chain]\rangle\right\} \qquad (1)$$

where U is the potential energy, n_c is the number of cellulose chains in the nanofibre and the single chain is simulated at the same boundary and thermodynamic conditions of the nanofibre. The brackets indicate ensemble average, that in the MD approach is equal to time average. The results of this section refer to nanofibres made of chains consisting of $n_r = 16$ pyranose rings, simulated at NVT ($T = 300$ K) conditions in an orthorhombic simulation cell with periodic boundary conditions (pbc) applied. Because of pbc, chains extend to infinity without any termination along their longitudinal axis.

In the limit of large samples, the average potential energy $\langle U\rangle$ becomes an extensive quantity. Therefore, to provide a property that can be compared for nanofibres of different n_c and n_r, the cohesive energy resulting from simulations has been divided by the number of ring in the whole system, equal to $n_c \times n_r$, see Tab. S1. It is apparent that, as expected, the cohesive energy per ring increases with increasing size, due to the decreasing weight of the surface energy on the sample cohesive energy.

While the variation of potential energy in forming a nanofibre or due to a change of temperature is unambiguously defined both in simulation and in experiments, and in principle could be measured by calorimetry, its subdivision in dispersion, Coulombic and intra-molecular contributions is somewhat ambiguous, since the different interactions are not completely disjoint and cannot be measured separately in experiments. For this reason we do not discuss in detail the role of different contributions to the cohesive energy, although they are easily determined by simulation. We limit ourselves to provide support to the statement that the Lennard-Jones (LJ) energy term, that contains dispersion and short range atom-atom repulsive interactions, is the major source of cohesion in cellulose nanofibres. The simulation results are given in Tab. S1, showing that U_{coh}^{LJ} represents at least 68 % of the total U_{coh}. According to the model, Coulomb terms account for about 24 % of U_{coh}, and intramolecular terms (stretching, bending and dihedral energies) account

TABLE S1: Cohesive energy at $T = 300$ K of cellulose crystal nanofibres consisting of 10, 18 and 61 chains. U_{coh} is expressed in kJ/mol per pyranose ring in the systems, whose number is $n_c \times n_r$. $\langle n_{HB}(interchain)\rangle$ is the average number of interchain H-bonds, again expressed per pyranose ring in the system. The average number of intra-chain H-bonds is more substantial, amounting to 1.45 ± 0.02, 1.51 ± 0.02, 1.54 ± 0.02 for the 10-, 18- and 61-chain nanofibres, respectively, but these bonds do not contribute to cohesion. These numbers are again per pyranose ring.

n_c	U_{coh}	U_{coh}^{LJ}	$\langle n_{HB}(interchain)\rangle$
10	41.8 ± 1	28.4 ± 1	0.27 ±0.02
18	48.5 ± 1	33.4 ± 1	0.38 ±0.02
61	55.0 ± 1	38.6 ± 1	0.44 ±0.02

for the rest.

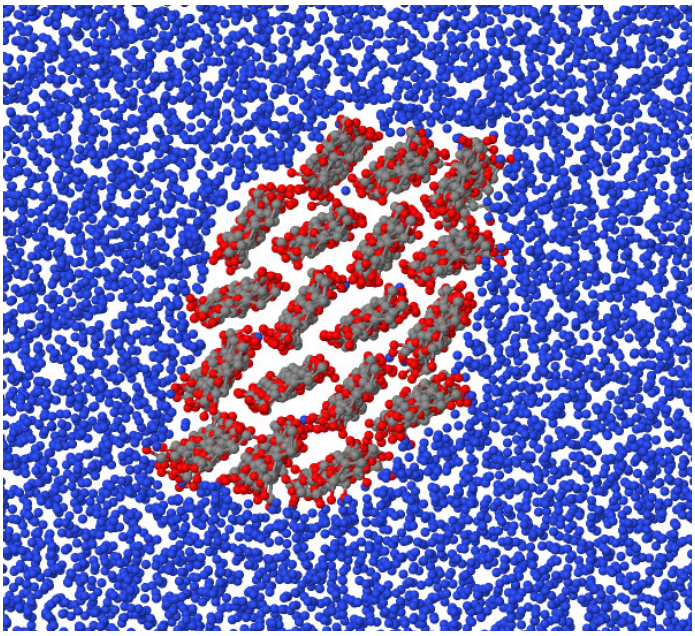

FIG. S2: Snapshot of the fully hydrated 18-chain crystal nanofibre seen along the longitudinal axis. Grey dots: C; red dots: O in cellulose; blue dots: O in water. Hydrogen is not shown.

In the model, the energy of H-bonds is represented as a combination of Coulomb (attractive) and LJ (primarily repulsive at the short distances of H-bonds) interactions, therefore their contribution to cohesion is contained in the terms already discussed. On the other hand, there is no simple and exact way to isolate the contribution of H-bonds to the cohesive energy, but only to make a very approximate estimate. In the present analysis, H-bonds are identified by geometric parameters only, as stated in Sec. III (a) of the main text. The average number of inter-chain H-bonds $\langle n_{HB}(interchain)\rangle$, again expressed per pyranose ring, is reported in in Tab. S1. Judging from the geometry, and especially from the oxygen-oxygen distance in the O-H—O triplet, these bonds are

of medium strength, and the energy of each bond is expected to be in the range of 36-40 kJ/mol. Therefore, their contribution to cohesive energy (referred to the single pyranose ring) ranges from 10 kJ/mol in the 1-chain nanofibre, to 16 kJ/mol in the 61-chain nanofibre.

Filling the simulation box with water at $P = 1$ atm. and $T = 300$ K using the *solvate* utility of Gromacs changes somewhat the number and distribution of H-bonds, decreasing the number of intra-chain H-bonds by 12 %, increasing the (relatively low) number of inter-chain H-bonds by 20 %, and introducing water-cellulose H-bonds into the picture. The number of these last H-bonds is proportional to the length of the nanofibres (equivalently, proportional to the number n_r of rings into each chain), and not proportional to the number n_c of chains in the nanofibre. In the case of the 18-chain crystal nanofibre, for instance, the average number of H-bonds in a stretch of the nanofibre 16-rings long is 240, corresponding to 15 H-bonds over the length of a pyranose ring (about 0.5 nm). The hydration state of the nanofibre surface can be appreciated in Fig. S2.

III. MORPHOLOGY AND ADSORPTION ENERGY OF A CALLOSE CHAIN ON PLANAR CRYSTALLOGRAPHIC SURFACES OF CELLULOSE

The morphology and adsorption energy of callose chains on the crystallographic surfaces of cellulose represent a basic information to rationalise the structure ordered and disordered assemblies of cellulose and callose chains.

Three crystal surfaces have been considered,[1] i.e., the (100), (010) and (110) whose geometry is illustrated in Fig. S3.

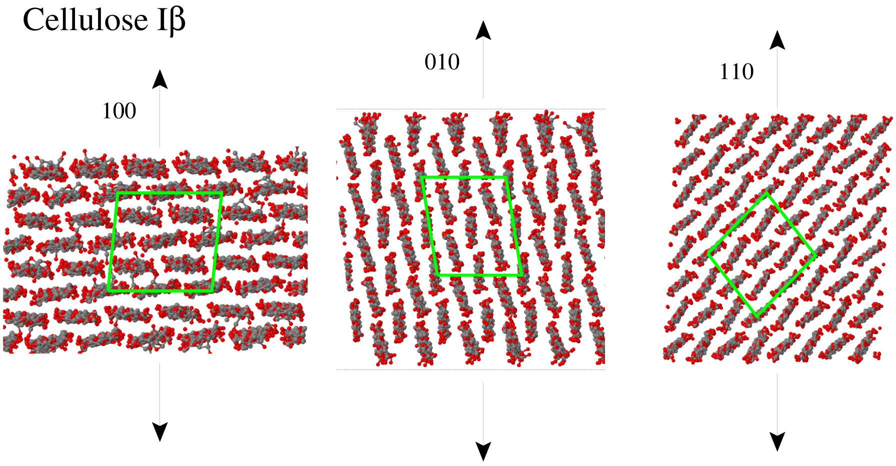

FIG. S3: Geometry of the three cellulose Iβ surfaces considered in the computation of the callose adsorption energy. The green quadrilateral shape identifies a unit cell of the Iβ crystal phase of cellulose. All samples have been equilibrated for about 100 ns.

A long equilibration at $T = 300$ K of a callose chain 48 pyranose rings long, lasting a few hundred ns, resulted in a coiled, globular configuration for this polymeric molecules, shown in Fig. S4 (a). This configuration and its average potential energy at $T = 300$ K have been taken as the reference to compute adsorption properties.

Given the similarity of of cellulose and callose, one could expect that a callose chain would unfold upon deposition on cellulose, growing the polysaccharide phase in a nearly epitaxial (Frank-van der Merwe) mode.[1] In MD simulation, however, unfolding could take a very long time, because of the low driving free energy and sluggish time evolution of a relatively complex polymeric species. For this reason, the deposition of callose in proximity of each surface has been simulated starting from two different callose configurations, i.e., the globular one (Fig. S4 (a)) resulting from the long equilibration, and a partially unfolded one (Fig. S4 (b)), resulting from a short (2 ns) equilibration of an extended callose chain.

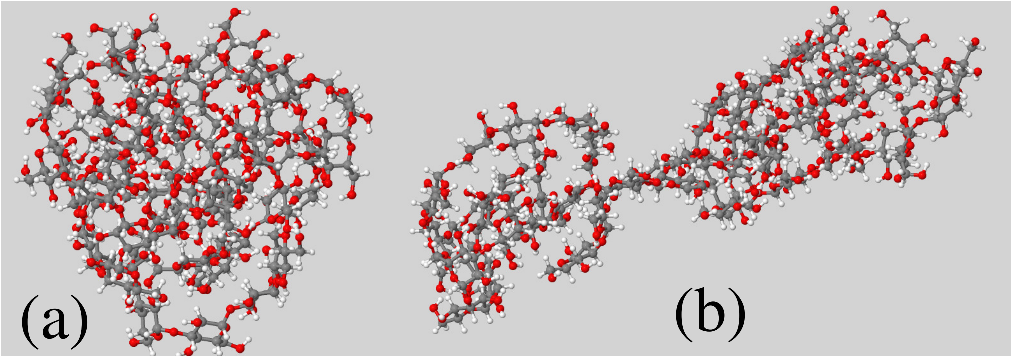

FIG. S4: Snapshot of a callose chain 48 pyranose rings. (a) long after a long equilibration (slightly more than 300 ns) at $T = 300$ K; (b) partly unfolded, after 2 ns equilibration starting from an extended configuration.

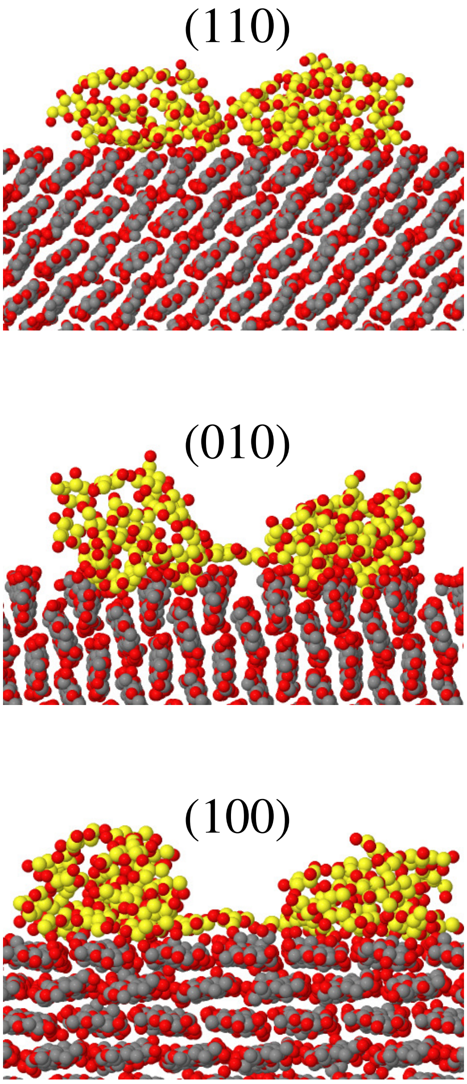

FIG. S5: Relaxed structure of a single callose chain (48 pyranose rings long) deposited on the three cellulose planar crystal surfaces shown in Fig. S3.

The results of the simulation confirm the very slow relaxation of the callose chain on the cellulose surface, since the final state of long (200 ns) relaxations still reflect the globular or partly unfolded geometry of the initial configuration.

For each of the simulated interfaces, the configurations of lowest average energy are systematically obtained upon starting from the partially unfolded geometry of callose (Fig. S4 (b)), although the adsorption energy obtained upon starting from the globular geometry are comparable. This energy ordering suggests that callose wets the planar cellulose surfaces, and only the slow kinetics of unfolding prevents a broad spreading of callose on cellulose. Of course, this assessment of

TABLE S2: Adsorption energy of a callose chain (48 pyranose rings long) adsorbed on three surfaces of the Iβ cellulose crystal. Energies are in kJ/mol for the entire chain. $\langle n_{HB} \rangle$ is the average number of H-bonds for the entire callose chain. The label ($ca \rightarrow ce$) indicated H-bonds donated by callose and accepted by cellulose; ($ce \rightarrow ca$) indicated H-bonds donated by cellulose and accepted by callose. The statistical error bar affects the last digit of the quantities listed in the table. Systematic errors due to incomplete equilibration are significantly larger.

Surface	E_{ads}	E_{ads}^{LJ}	$\langle n_{HB} \rangle$ ($ca \rightarrow ce$)	$\langle n_{HB} \rangle$ ($ce \rightarrow ca$)
(100)	-412	-384	5.55	3.5
(010)	-546	-478	9.2	13.3
(110)	-402	-335	7.2	9.0

relative stability is only approximate, both because the samples do not relax to their unique most stable morphology, and because thermodynamic stability relies on free energy and not on potential energy minimisation. However, at room temperature the role of entropy is not very significant, and the scale of adsorption energy is assumed to reflect the relative stabilities of configurations.

The inability of the 200 ns simulation runs to attain equilibrium is confirmed by the fact that the final potential energies (averaged over the last 20 ns of simulation) of repeated deposition simulations are rather scattered, suggesting that the uncertainty of the adsorption energies is of the order of 50 kJ/mol.

Keeping in mind these limitations, the configuration of lowest potential energy on the three surfaces of interest are shown in Fig. S5, while the adsorption energy at 300 K is reported in Tab. S2. This quantity is defined in the usual way[1] as $E_{ads} = \langle U[substrate + adsorbate] \rangle - \langle U[substrate] \rangle - \langle U[adsorbate] \rangle$, hence negative values are associated with stable adsorption. The adsorption energies listed in Tab. S2 refer to the whole callose chain and not to the single pyranose ring, since E_{ads} is not likely to be an extensive quantity, i.e., it is not linearly proportional to the number of pyranose rings n_r in the callose chain. The number of cellulose-callose H-bonds is sizeable, especially for the (010) and (110) surfaces. Also in this case, the O—O distance corresponds to H-bonds of medium strength. The contribution of H-bonding to E_{ads}, however, is not necessarily proportional to the number of cellulose-callose H-bonds, since the formation of these bonds is partially compensated by the breaking of callose-callose and cellulose-cellulose H-bonds. This observation is supported by the fact that the contribution of LJ energy alone accounts for most of E_{ads} on all simulated surfaces.

Within the large estimated error bar, one can observe that adhesion on the compact (100) and (110) surfaces is weaker than on the open (010) surface. The structures shown in Fig. S5 suggest that stronger adhesion is due to the penetration of callose in the grooves of the (010)

surface. Hydrogen bonding might contribute significantly to this enhanced adhesion, as shown by the average number n_{HB} of hydrogen bonds linking callose and cellulose given in Tab. S2.

IV. ADSORPTION ENERGY OF CALLOSE ON CELLULOSE NANOFIBRES

The computation of the adsorption energy of callose on cellulose crystalline nanofibres is more challenging than in the case of planar surfaces, since the combination of different crystal facets in nanofibres provides a wider variety of adsorption configurations. Given the slow kinetics of callose on cellulose, MD simulations lasting $\sim 10^2$ ns are not enough to approach equilibrium, remaining trapped in metastable configurations depending on the starting geometry. Then, the adsorption energies resulting from different (simulated) depositions of callose on nanofibres are rather scattered, as already observed in the case of adsorption on planar surfaces. In this case, however, the lowest energy results are obtained starting from the folded (globular) configuration, and the systematic difference from those obtained starting from unfolded callose is significant. Moreover, as expected, the adsorption energy increases with increasing size of the nanofibre.

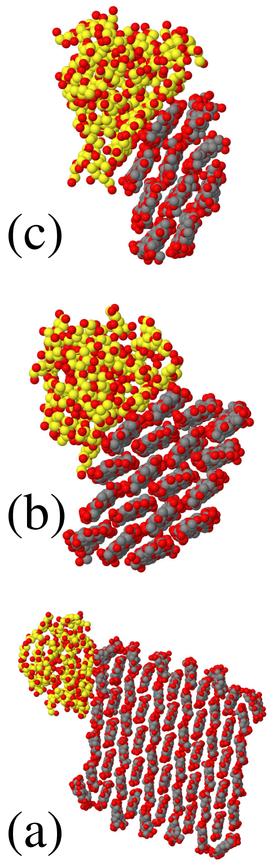

FIG. S6: Relaxed structure of a single callose chain (48 pyranose rings long) deposited on cellulose crystal nanofibres consisting of: (a) 61-chains; (b) 18 chains; (c) 10 chains.

These results suggest that, first of all, the close contact of callose with the combination of different surfaces requires a more substantial unfolding, whose energy cost is not sufficiently compensated by the defective contact of callose and cellulose crystal faces. The strengthening of adhesion with increasing nanofibre size could have been expected, because larger nanofibres present larger flat facets, on which callose needs less deformation/unfolding to extend its adhesion area.‘

The structures of lowest energy are shown in Fig. S6, while the corresponding adsorption energies are in Tab. S3. As before, the adsorption energy is computed as the difference between the average potential energy at $T = 300$ K of the combined callose plus nanofibre sample minus the energy of the two separate parts, i.e., the average potential energy of the nanofibre and of the folded chain.

Although the results concern samples that are not at equilibrium, the adsorption energies in the range of hundreds of kJ/mol per callose molecule confirm that the sticking of callose on cellulose is irreversible, and the relaxation of the combined structures is severely limited by a slow kinetics.

TABLE S3: Adsorption energy of a callose chain (48 pyranose rings long) adsorbed on three crystal nanofibres made of n_s cellulose chains. Energies are in kJ/mol for the entire chain. The label ($ca \rightarrow ce$) indicated H-bonds donated by callose and accepted by cellulose; ($ce \rightarrow ca$) indicated H-bonds donated by cellulose and accepted by callose. The number of significant digits reflects the statistical error along the simulated trajectories. Larger uncertainties of the order of 50 kJ/mol are associated to the dependence of the result on the starting point.

n_c	E_{ads}	E_{ads}^{LJ}	$\langle n_{HB} \rangle$ ($ca \rightarrow ce$)	$\langle n_{HB} \rangle$ ($ce \rightarrow ca$)
10	-295	-286	2.75	1.70
18	-384	-329	2.10	1.40
61	-482	-398	2.20	1.70

More equilibrated results could be obtained using accelerated MD methods, but the hydrogels investigated in experiments are equally out of equilibrium, and the exploration of structures and energies carried out in the present study might be closer to reality than a description based on equilibrium and accelerated sampling.

The globular geometry of callose even on the smallest nanofibre (10-chains) suggests that the open configurations seen in the hydrogel structures, showing callose chains joining different nanofibres and single chains are metastable structural motifs, resulting from the sticking of extended callose chains to different adsorption points during relaxation.

Also in this case, the Lennard-Jones energy term represents the major contribution to adsorption energy.

V. SUPPORTING INFORMATION ON THE X-RAY DIFFRACTION ANALYSIS

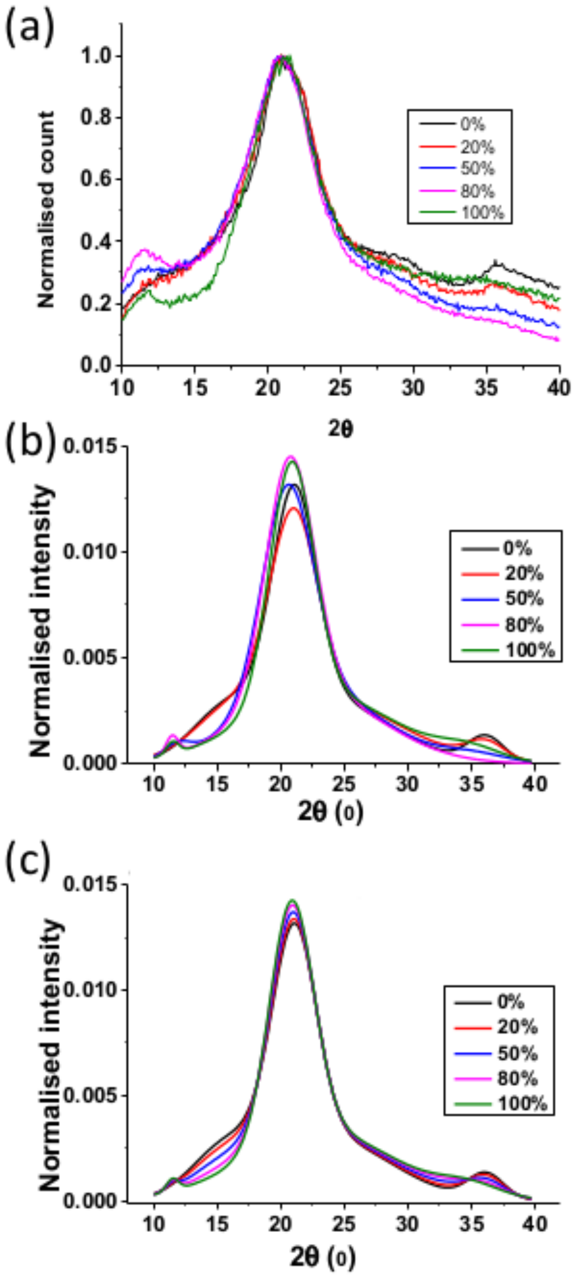

FIG. S7: X-ray diffractograms for dried hydrogels. The graphs show the curves obtained after normalizing counts (a), normalizing according to area (b), and predicted theoretical mixing (c) the signal intensity of the dried hydrogels containing 0, 20, 50, 80 and 100 % of pachyman in relation to cellulose in a 10 % wt total polysaccharide.

VI. THE DIFFUSION OF WATER IN THE SIMULATED HYDROGELS

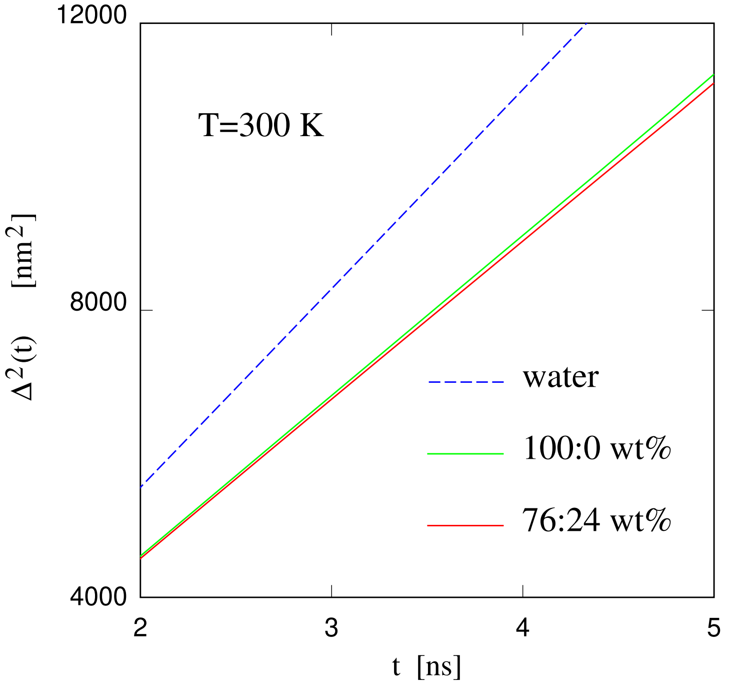

FIG. S8: Mean square displacement as a function of time of the OW oxygen atom of water in three simulated samples.

The diffusion properties of water in the 100:0, ..., 76:24 hydrogel samples have been determined by computing the time dependence of the mean square displacement $\Delta^2(t)$ of the OW water oxygen atoms, averaged over the last 20 ns of the simulation trajectories. The linear coefficient $p = \lim_{t\to\infty} \Delta^2(t)/t$ gives the self-diffusion coefficient $D_W = p/6$, according to the well known Einstein relation. The results for $\Delta^2(t)$ at $T = 300$ K in pure water and in the two hydrogel of 100:0 and 76:0 cellulose-callose concentration are shown in Fig. S8. The results for the two intermediate concentrations have been omitted because the curves are very close to each other. The statistical error bar (estimated for D_W at ± 0.01 cm^2/s) is very low since the data for $\Delta^2(t)$ are averaged over $\sim 500,000$ water molecules and over ~ 100 starting points t_0 in the estimation of $\langle | \, \mathbf{r}(t_0 + t) - \mathbf{r}(t_0) \, |^2 \rangle$. Also in this case, the systematic error is much larger, being due to the overestimation by the SPC model of the diffusion constant of water, $D_W^{SPC} = 4.62 \pm 0.01$ cm^2/s versus the experimental $D_W^{exp} = 3.65 \pm 0.01$ cm^2/s at $T = 300$K. This error, however, does not prevent the accurate determination of trends as a function of cellulose-callose relative concentration.

The full set of computed diffusion coefficients is: $D_W^{SPC} = 4.62$ cm^2/s in pure water; $D_W = 3.75$ cm^2/s in 100:0; $D_W = 3.74$ cm^2/s in 92:8; $D_W = 3.73$ cm^2/s in 84:16; $D_W = 3.70$ cm^2/s in 76:24. The error bar on D_W is ± 0.01 cm^2/s in all cases.

VII. WATER ADSORPTION ON THE BUNDLE OF SEVEN CELLULOSE NANOFIBRES (18-CHAINS EACH) WITH NO CALLOSE

The adsorption of water on the bundle made of seven nanofibres (18-chains each) briefly introduced in Sec. IV F of the main text (see Fig. 11) has been investigated by simulation to provide a comparison for the results on the water penetration in bundles made of cellulose and callose.

The hydrophobic character of the cellulose bundle surface is apparent from the snapshot shown in Fig. S9. The water islands on the surface represent a clear example of Vollmer-Weber growth of the adsorbate on the surface.

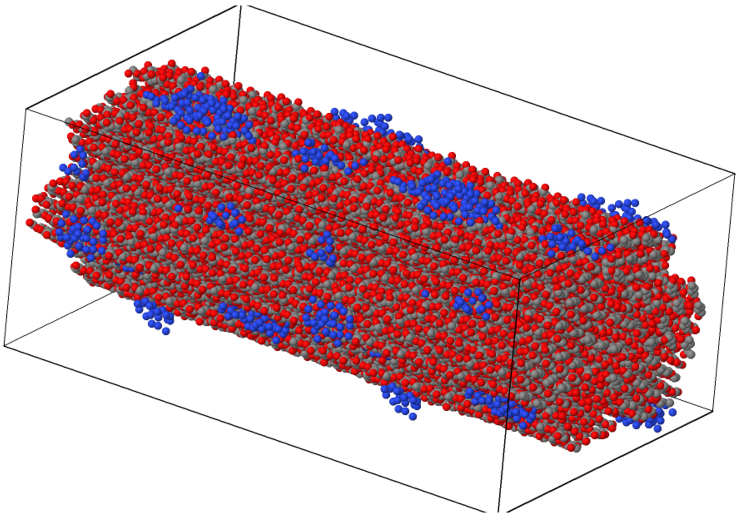

FIG. S9: Snapshot of the bundle of 18-chain cellulose nanofibres without callose and with 480 water molecules adsorbed on its surface. The simulates sample has been replicated twice along its axis. The snapshot show the Volmer-Weber growth of water on cellulose nanocrystals.

A view of the water distribution seen from the axis of the bundle shows that water tends to accumulate in the crevices formed at the junction of different nanofibres in the bundle, but does not penetrate deep into the structure.

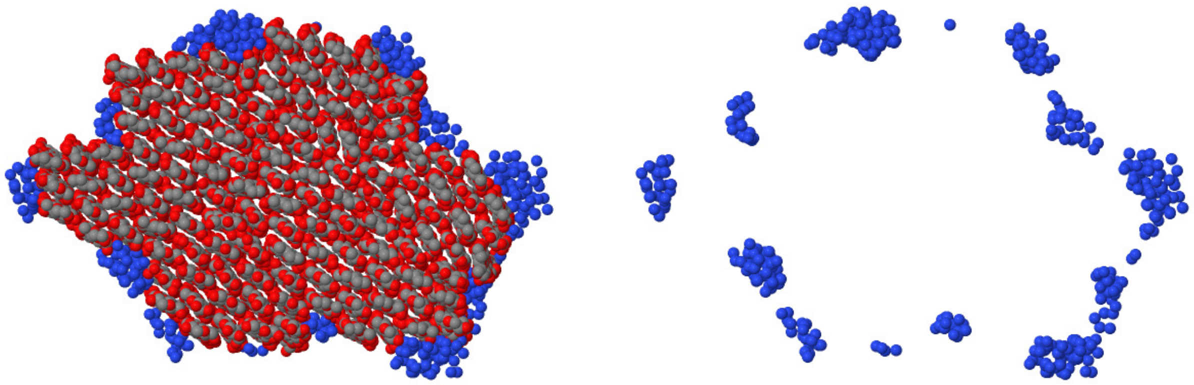

FIG. S10: The same bundle of Fig. S9 seen from the axis to show the radial distribution of water.

The previous observation is confirmed by the result of the simulation of the bundle in an extended distribution of water at normal conditions. This last sample contains 59373 water molecules.

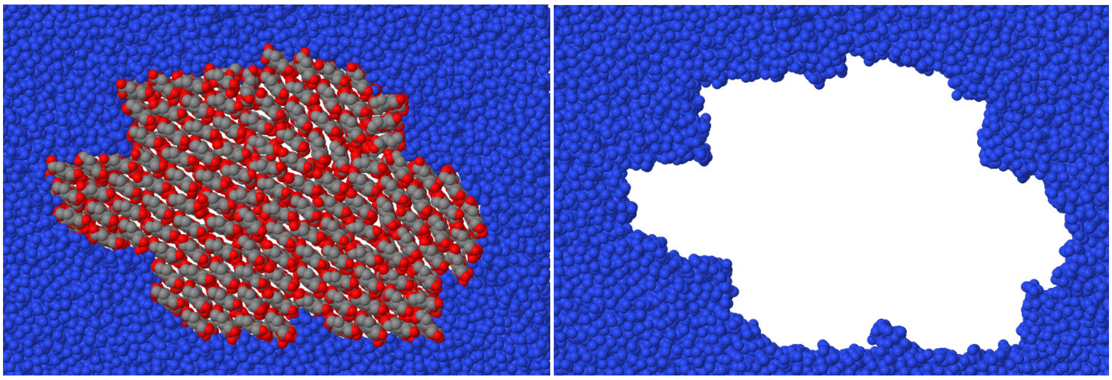

FIG. S11: The same bundle of Fig. S9 in bulk water. The picture represents part of the simulated sample.

VIII. SUPPLEMENTAL INFORMATION ON MASS RECOVERY AFTER REHYDRATION

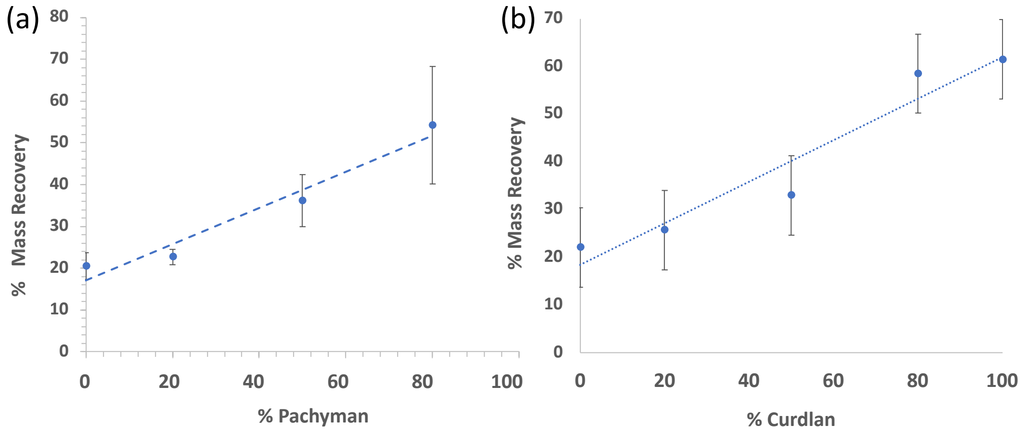

FIG. S12: Adding either pachyman or curdlan improves mass recovery after gel rehydration. Graphs show mass recovery after full rehydration of oven-dried hydrogels containing different concentrations of Pachyman (a) or Curdlan (b). Linear regression curves are shown to guide the eye. Error bars are standard deviation.

IX. SUPPLEMENTAL INFORMATION ON THE DYE UPTAKE CAPACITY

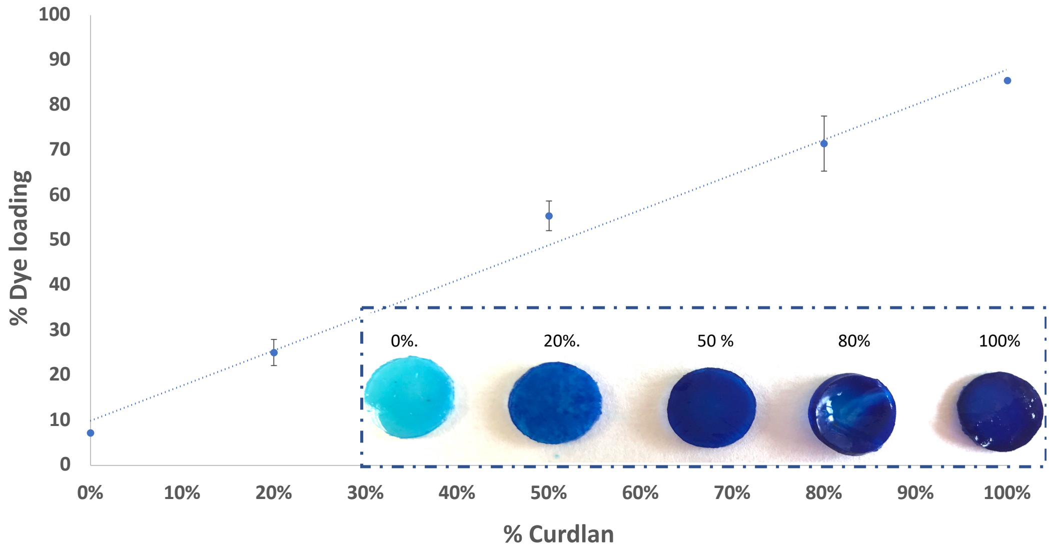

FIG. S13: Curdlan increases the dye uptake capacity of cellulose. The graph shows methylene blue loading after 24h immersion of hydrogels containing cellulose and curdlan (x axis shows the percentage of Curdlan in each hydrogel). Error bars are standard deviation. Differences in dye loading are evident in the images showed in the graph.

The hydrogels prepared as described in the main text were submerged in the methylene blue solution (250 μg/ml) for 24h in a sealed bottle. After loading, the hydrogel was removed and the absorbance of the remaining solution was measured to calculate dye loading. The mass of the dye loaded were calculated using the calibration curve linear regression as: $y = 0.1913x - 0.0273$; $R^2 = 0.9998$ (where y= Absorbance at 665nm; X= dye mass loading).

X. SUPPLEMENTARY INFORMATION ON THE DYE RELEASE RATE IN HYDROGELS

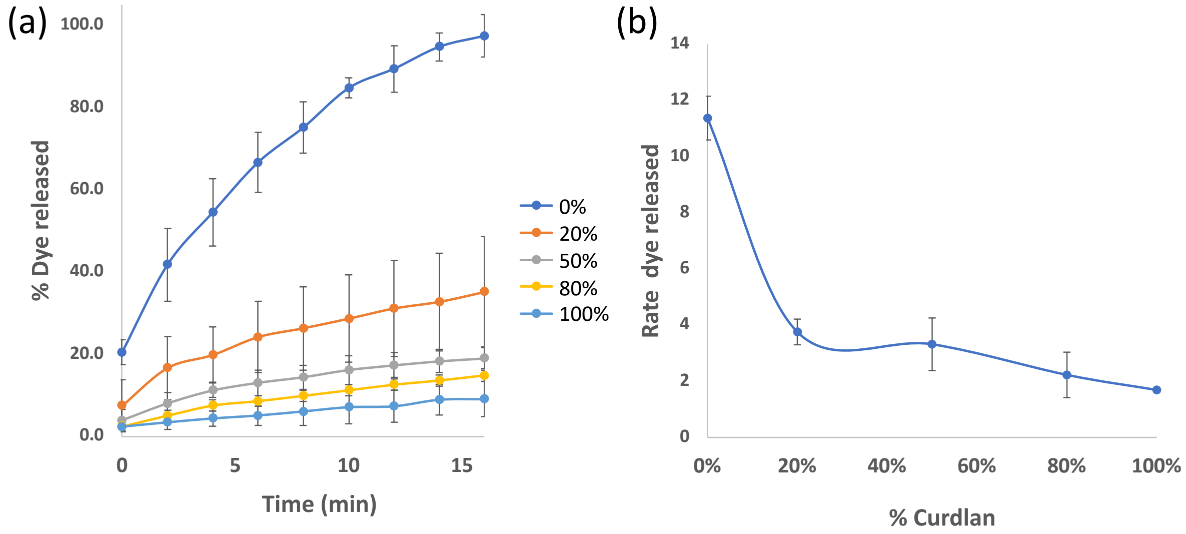

FIG. S14: Hydrogels containing curdlan (commercial β-1,3 glucan) display slower dye release compared to cellulose gels. Graphs show (a) percentage of dye released measured at 2min intervals during a total of 16 min for different percentage of curdlan. (b) The rate of the dye released as a function of percentage of curdlan in the gel. Error bars are standard deviation.

To calculate dye release, the loaded hydrogel was submerged in 10^4 ml of fresh water. The absorbance of the aqueous solution was measured at 2 minute intervals during 16 min. The release was calculated using absorbance and the calibration curve described above. The percentage of the dye release was calculated taking as a reference the dye loaded on the gel calculated before. The percentage of the dye release was represented and the slope of the linear part of the curve was referred to as rate release.

XI. THE EFFECT OF [EMIM][OAC] CONTAMINATION, THE AMOUNT OF AMORPHOUS CELLULOSE, THE SIZE OF THE NANOFIBRES, AND OF CHAIN TERMINATIONS ON HYDROGEL STRUCTURE

The simulation of cellulose and callose hydrogels has been supplemented with the investigation of a sample in which [emim][OAc] is dissolved at low concentration in water. The ability of ILs to dissolve cellulose nanofibres has been known and discussed for a relatively long time.[2,3] Since [emim][OAc] is used to dissolve cellulose nanofibres before being replaced by water in the preparation of the hydrogels, the aim of these simulations is to assess the relative affinity of the ions for cellulose, callose and water, which could affect the hydrogel formation. To this aim, water has been removed from the equilibrated 76:24 sample. Then, 264 ion pairs have been added at random positions in the wide cavities left in the dry polysaccharide sample, rejecting the insertions that lead to a superposition of atoms to within their van der Waals radii. Finally, the sample has been hydrated using again the *solvate* utility of Gromacs, resulting in a fairly homogeneous distribution of ions across the system. The sample has been equilibrated for slightly more than 100 ns by MD at NPT conditions, sufficient to produce a stationary distribution of ions, but probably not to change the hydrogel structure. Analysis of trajectories show that about 70 % of the ions are adsorbed on the nanofibre surfaces, or have a short contact (distance < 3.6Å) with callose or cellulose single chains, with no obvious preference for either species. The remaining 30 % of the ions remain in solution. Moreover, at these low concentrations the $[\text{emim}]^+$and $[\text{OAc}]^-$ ions in solution tend to remain dissociated, diffusing fairly easily and independently in water. The $[\text{emim}]^+$ cations, in particular, interact with the polysaccharide chains primarily by dispersion forces, while their fair solubility is due to their small size and to their charge gaining free energy through electrostatic screening by water. The $[\text{OAc}]^-$ anion is able to accept multiple HBs through its -COO^- moiety. In the simulation, the majority of the HBs accepted by $[\text{OAc}]^-$ are donated by water, and to a lesser extent by cellulose and callose. In each snapshot, several anions appear to be H-bonded at the same time to water and polysaccharides. Both $[\text{emim}]^+$ and $[\text{OAc}]^-$, therefore, present specific mechanisms to bind simultaneously to water and cellulose/callose, thus [emim][OAc] represents a good surfactant favouring the homogeneous distribution of cellulose and callose in the solution while the IL is being replaced by water. Moreover, the association of ions to the polysaccharide species has a dynamic equilibrium character, with frequent exchanges between the adsorbed and solvated ion populations. Also this aspect favours the progressive replacement of IL with water during the hydrogel preparation.

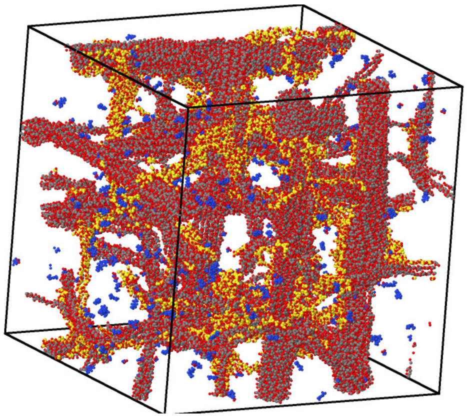

FIG. S15: Snapshot from the simulation of sample 76:24 with the addition of 264 $[emim]^{+}$ and $[OAc]^{-}$ ion pairs. All ions are painted blue. The carbon atoms of callose has been painted yellow. Cellulose is represented by black (C) and red (O) dots.

Further simulations have been carried out for a variant of the basic hydrogel model discussed up to this point, with the aim of investigating the dependence of the number and distribution of links on the presence of amorphous cellulose, on the size of the crystal nanofibres, and on the presence of chain terminations. In this second model, cellulose chains are represented as infinitely extended through the usage of pbc and no chain terminations. Moreover, cellulose is present only in the nanocrystalline fibre phase, consisting of a number (from 9 to 12) of 18-chain nanofibres. Callose chains, instead, are of finite length and terminated as before by complementary -OH and -H groups. The relation between the new (Panel (a)) and the previous (Panel (b)) model is illustrated in Fig. S16. In some respects, the new model, in which cellulose chains are much longer than the simulation scale, and are only present as 18-chain crystalline nanofibres, might correspond better to the structure of cellulose and callose mixtures in biological systems, in which the amorphous phase of cellulose is not present or not relevant, and the structure/size of the native nanofibres is more consistently retained.

At the beginning, a sample closely resembling 100:0 of the main simulations is prepared by inserting twelve nanofibres in the simulation cell, divided in three groups of four nanofibres aligned with each of the Cartesian axes. Further samples are obtained by replacing one nanofibre with 18 callose single chains, initially distributed at random in the simulation cell. Nanofibres are replaced one per direction in turn. Each cellulose and callose chain is 48 pyranose rings long therefore the replacement does not change the total polysaccharide concentration. As before, the preparation stage is completed by filling the simulation box with water, using the *solvate* utility of Gromacs and resulting in samples of about 1.6×10^{6} atoms. The samples created in this way are very similar to

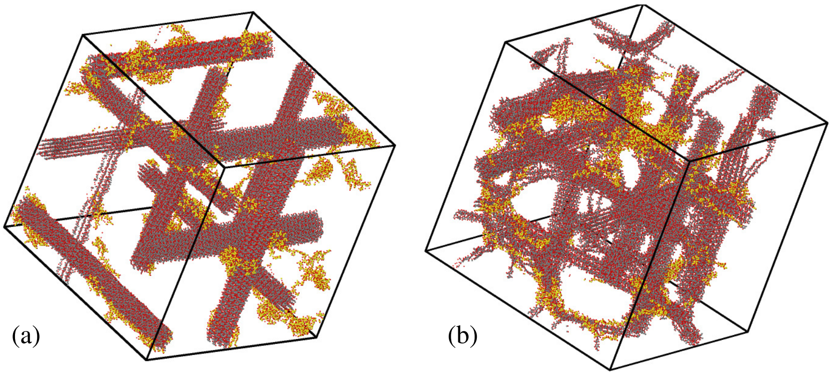

FIG. S16: Comparison of cellulose-callose in water structures used in the simulations. Panel (a): sample in which all cellulose is present in the form of crystalline nanofibres. Panel (b): sample in which 40 wt% of cellulose is present as single chains. The composition of the two samples is the same, corresponding to that of 84:16 in Tab. I of the main text. Black dots: C in cellulose; yellow dots: C in callose; red dots: oxygen in cellulose and callose. Hydrogen atoms and water not shown.

the 100:0, ..., 76:24 samples of the main simulation, but the two sets of models evolve along rather different paths. First of all, because of pbc, the alignment of cellulose nanofibres along the axes is strictly conserved during the simulation, and this is the most apparent effect of removing the cellulose chain terminations extending nanofibres through pbc. As before, the finite callose chains have a variable orientation, and form links among the cellulose nanofibres. However, lacking the support of the single cellulose chains, callose chains are more compact, having an average end-to-end separation $\langle l \rangle = 6.6 \pm 0.2$ nm, against the $\langle l \rangle = 10.3 \pm 0.2$ nm in the previous case. Again, lacking the contribution of single cellulose chains, the total number of links joining crystalline nanofibres is much less than in the previous model, and below the relative cellulose:callose concentration of 84 : 16 wt% (with respect to the total 10 wt% polysaccharide in the sample) the polysaccharide sub-system can be divided into disjoint units. Only at callose content above the 84 : 16 wt% the simulated sample is fully connected and might represent a hydrogel phase. These statements on connectivity depend on the size of the sample and on the length of the connecting chains (callose), therefore cannot be taken as general. Nevertheless, these observations confirm that, as expected, samples made of cellulose crystalline nanofibres and no amorphous cellulose fraction, require more callose linkers to give origin to a stable hydrogel. The water-water structure factor $S_{WW}(k)$ computed for this second model is very similar to the one computed with the previous hydrogel model confirming that the large peak at low-k reflects primarily the correlation in the distribution of nanofibres, with only a low dependence on the fibre size and on the presence of

the amorphous phase. On the other hand, the $S_{RR}(k)$ computed for the pyranose rings shows significant differences at all k in the two cases, reflecting its dependence on the size and crystal ordering of the nanofibres. Identification of all H-bonds in the sample show that the number of H-bonds linking cellulose and water is reduced roughly by half, since chains incorporated in the nanofibre are isolated from water, or at least partially engaged in cellulose-cellulose H-bonding. The number (per chain) of water-callose H-bonds, instead, is nearly unchanged from the previous case. The decrease of links among nanofibres, as well as the reduced hydration of cellulose, suggest that the single cellulose chains representing the amorphous fraction of the experimental samples play a major role in stabilising the homogeneous distribution of polysaccharides in water and in the formation of hydrogels.

[1] Zangwill, A. *Physics at Surfaces*, Cambridge University Press, Cambridge (1988).

[2] Cho, H. M.; Gross, A. S.; Chu, J.-W. Dissecting force interactions in cellulose deconstruction reveals the required solvent versatility for overcoming biomass recalcitrance. *J. Am. Chem. Soc.* **2011**, *133*, 14033.

[3] Yuan, X.; Cheng, G. From cellulose fibrils to single chains: understanding cellulose dissolution in ionic liquids. *Phys. Chem. Chem. Phys.* **2015**, *17*, 31592-31607.
